# A review of the diagnosis and geographical distribution of the recently described flea toad *Brachycephalus sulfuratus* in relation to *B. hermogenesi* (Anura: Brachycephalidae)

**DOI:** 10.7717/peerj.10983

**Published:** 2021-03-04

**Authors:** Marcos R. Bornschein, Luiz Fernando Ribeiro, Larissa Teixeira, Ricardo Belmonte-Lopes, Leonardo Amaral de Moraes, Leandro Corrêa, Giovanni Nachtigall Maurício, Júnior Nadaline, Marcio R. Pie

**Affiliations:** 1 Departamento de Ciências Biológicas e Ambientais, Universidade Estadual Paulista, São Vicente, São Paulo, Brazil; 2 Mater Natura - Instituto de Estudos Ambientais, Curitiba, Paraná, Brazil; 3 Programa de Pós-Graduação em Biologia Animal, Universidade Federal de Pelotas, Pelotas, Rio Grande do Sul, Brazil; 4 Departamento de Zoologia, Universidade Federal do Paraná, Curitiba, Paraná, Brazil

**Keywords:** *Brachycephalus didactylus* group, Advertisement call, Morphology, Taxonomy, Diagnose, Guapiara lineament, Biogeography, Sympatry, Note-centered approach, Cryptic species

## Abstract

**Background:**

The flea toad *Brachycephalus sulfuratus* was recently described from southeastern and southern Brazil. In its description, the authors overlooked previous records of flea toads that had been identified as “*Brachycephalus* sp. nov.” and *B. hermogenesi* occurring in the same regions, which could suggest the possibility of up to three flea toads coexisting in southern Brazil. In addition, *B. sulfuratus* is characterized by substantial phenotypic variability, to an extent that compromises its current diagnosis with respect to its congener *B. hermogenesi*. Therefore, the current state-of-affairs regarding the geographical distribution of these two species and the identification of previously known populations is hitherto uncertain. Our goals are to reassess previous records of flea toads attributable to *B. hermogenesi*, *B. sulfuratus* and “*Brachycephalus* sp. nov.”, considering the description of *B. sulfuratus*, and to review the diagnosis of *B. sulfuratus*.

**Methods:**

A critical analysis of the species identity of flea toad specimens attributable to *B. hermogenesi*, *B. sulfuratus*, or to a potentially undescribed species from southeastern and southern Brazil was based either on the analysis of morphology or on their advertisement calls. These analyses include our independent examinations of specimens and, when not possible, examinations of published descriptions. To allow for a consistent comparison of advertisement calls between *B. hermogenesi* and *B. sulfuratus*, we made recordings of both species, including in the type locality of the former.

**Results:**

We found that morphological and call characters originally proposed as diagnostic for *B. sulfuratus* in relation to *B. hermogenesi* vary intraspecifically. Live individuals with ventral yellow spots correspond to *B. sulfuratus*; individuals without yellow spots can be either *B. sulfuratus* or *B. hermogenesi*. In preservative, they are indistinguishable. Previous records of *Brachycephalus* sp. nov. correspond to *B. sulfuratus*. We propose that the reduced number of notes per call and the presence of only isolated notes in the call of *B. sulfuratus*, as opposed to a high number of notes per call with isolated notes and note groups in the call of *B. hermogenesi*, as the only diagnostic characters between them. Regarding their distributions and based in our assessment, only *B. sulfuratus* occurs in southern Brazil, without any overlap with *B. hermogenesi*. There is a narrow gap between the distributions of these species around the southeast of the city of São Paulo. Our revision also revealed that some records previously attributed to *B. hermogenesi* in Rio de Janeiro and north São Paulo represent a distinct, unidentified flea toad that is not *B. sulfuratus*. Both species occur side by side in Corcovado, São Paulo, a locality from where five paratypes of *B. hermogenesi* were obtained. Biogeographic events that might have led to vicariance between *B. hermogenesi* and *B. sulfuratus* are discussed.

## Introduction

The genus *Brachycephalus* Fitzinger, 1826 includes 36 small diurnal anuran species that live in the leaf litter across the Brazilian Atlantic Rainforest (Bornschein, Pie & Teixeira, 2019). Most species present small geographic distributions, restricted to one or a few adjacent mountaintops (Pie et al., 2013; Bornschein et al., 2016a; Bornschein, Pie & Teixeira, 2019). *Brachycephalus* has been divided in three phenetic groups, based on body shape and presence/absence of dermal co-ossification (Ribeiro et al., 2015), and presence/absence of *linea masculinea* (Pie et al., 2018b): the *B. ephippium* group, with 12 species distributed from Espírito Santo and Minas Gerais south to São Paulo, southeastern Brazil (Bornschein, Pie & Teixeira, 2019); the *B. pernix* group, with 19 species distributed in Paraná and Santa Catarina, southern Brazil (Bornschein, Pie & Teixeira, 2019); and the *B. didactylus* group, with four species commonly known as flea toads and distributed throughout much the Atlantic Forest of Brazil, from Bahia to Santa Catarina, northeastern, southeastern, and southern Brazil (Bornschein, Pie & Teixeira, 2019). Members of the *B. didactylus* species group (sensu Ribeiro et al., 2015; Pie et al., 2018a; Bornschein, Pie & Teixeira, 2019) are distinguished by their leptodactyliform body shape and the absence of dermal ossification and absence of *linea masculinea*. The *B. ephippium* species group includes species with bufoniform body shape, presence of dermal ossification and absence of *linea masculinea*, and, finally, the *B. pernix* species group includes species equally with bufoniform body shape but without dermal ossification and with *linea masculinea* (Ribeiro et al., 2015; Pie et al., 2018a).

The first described flea toad species was *B. didactylus*, in 1971 (Izecksohn, 1971) as the only member of a new genus, *Psyllophryne*. The second flea toad species, *B. hermogenesi*, was described nearly three decades later, in 1998 (Giaretta & Sawaya, 1998), at the time as the second species of the genus *Psyllophryne*. This genus was then synonymized in favor of *Brachycephalus* when it was discovered that this genus also had an omosternum, whose presence until then exclusive in *Psyllophryne* diagnosed that genus in relation to *Brachycephalus* (Kaplan, 2002). Recently, other two flea toads were described, namely *B. pulex* (Napoli et al., 2011) and *B. sulfuratus* (Condez et al., 2016). Only recently have flea toads been recorded in southern Brazil. The first records were of *B. hermogenesi* to the Reserva Particular do Patrimônio Natural Salto Morato (RPPNSM), municipality of Guaraqueçaba, in the northern coast of Paraná (Pereira et al., 2010; Santos-Pereira et al., 2011) and at Colônia Castelhanos, municipality of Guaratuba, in southern Paraná, initially as “*Brachycephalus* aff. *hermogenesi*” (Cunha, Oliveira & Hartmann, 2010) and later as “*B. hermogenesi*” (Oliveira et al., 2011). Shortly thereafter, Pie et al. (2013) published 14 localities of a flea toad identified as “*Brachycephalus* sp. nov. 1”, from Paraná and Santa Catarina. These authors also reidentified the record from Colônia Castelhanos as “*Brachycephalus* sp. nov. 1”. Occurrences from RPPNSM of Pereira et al. (2010) and Santos-Pereira et al. (2011) were overlooked by Pie et al. (2013). Later, Bornschein et al. (2016a) compiled 18 localities of a flea toad as *Brachycephalus* sp. 1., including the 14 localities of Pie et al. (2013) treated as “*Brachycephalus* sp. nov. 1”. Bornschein et al. (2016a) also reidentified previous records of the flea toad of the RPPNSM and Colônia Castelhanos as *Brachycephalus* sp. 1.

After these discoveries, the flea toad *B. sulfuratus* was described in 2016 based on a series of 28 specimens distributed from southern São Paulo to northern Santa Catarina (Condez et al., 2016). However. these authors did not take into account the information available in Pie et al. (2013) and Bornschein et al. (2016a). Rather, Condez et al. (2016) only considered the presence of the flea toad *B. hermogenesi* in Paraná, based on Oliveira et al. (2011). However, the voucher specimen of Oliveira et al. (2011), a single specimen deposited in the Museu de História Natural, Universidade Estadual de Campinas, Campinas (ZUEC 16602), was reidentified by Condez et al. (2016) as *B. sulfuratus*, whereas the remaining records of *B. hermogenesi* in Paraná, from Pereira et al. (2010) and Santos-Pereira et al. (2011), were not considered by Condez et al. (2016).

The absence of a nomenclatural review of records of flea toads in southern Brazil can be evidenced by the fact that a single location in Santa Catarina, called Castelo dos Bugres, was recorded as harboring specimens identified as “*Brachycephalus* sp. nov. 1” (Pie et al., 2013), or *Brachycephalus* sp. 1. (Bornschein et al., 2016a) and *B. sulfuratus* (Condez et al. (2016). No analysis has been carried out to ensure that the unidentified species represents *B. sulfuratus*, so that the uncertainty in the identification of some important occurrence records seems to indicate three possible scenarios. First, one could envision that potentially there are three similar species of flea toads in Paraná and Santa Catarina, southern Brazil, namely *B. hermogenesi* (Pereira et al., 2010; Santos-Pereira et al., 2011), *Brachycephalus* sp. (Pie et al., 2013; Bornschein et al., 2016a) and *B. sulfuratus* (Condez et al., 2016). Second, records of *B. hermogenesi* in southern Brazil could be erroneous, given that some of these records (Cunha, Oliveira & Hartmann, 2010; Oliveira et al., 2011) were assigned to *B. sulfuratus* or “*Brachycephalus* sp. nov.” (Pie et al., 2013; Condez et al., 2016), leading to an expectation that two species might occur in these regions (*B. sulfuratus* and *Brachycephalus* sp.). Third, if the unidentified species of Pie et al. (2013) and Bornschein et al. (2016a) is conspecific of *B. sulfuratus*, there could be a single species of flea toad in southern Brazil (*B. sulfuratus*).

Recently, Bornschein, Pie & Teixeira (2019) reviewed the available occurrence records of flea toads from southeastern and southern Brazil and reverted most of the records of “*Brachycephalus* sp. nov. 1” (Pie et al., 2013), “*Brachycephalus* sp. 1” (Bornschein et al., 2016a), and *B. hermogenesi* from southern Brazil (Pereira et al., 2010; Santos-Pereira et al., 2011, 2016) in favor of *B. sulfuratus*. Some records that could not be adequately reassessed by Bornschein, Pie & Teixeira (2019) were reverted to “*Brachycephalus* sp. cf. *B. sulfuratus*”, including the records of *B. hermogenesi* from Cunha, Oliveira & Hartmann (2010) and Oliveira et al. (2011). Bornschein, Pie & Teixeira (2019) disregarded the possibility of a third unnamed species of flea toad in southern Brazil, but one question remains: the proper identification of *B. sulfuratus* and *B. hermogenesi*. In this sense, the identification criteria used by Bornschein, Pie & Teixeira (2019) to reevaluate the records of flea toads were not indicated. In addition, there may still be uncertainty in the identification of flea toads by other authors, as records of *B. hermogenesi* in southern Brazil continue to be published (Santos-Pereira et al., 2016; Santos-Pereira, Pombal & Rocha, 2018; Leivas et al., 2018). Given this uncertainty, the aim of this study is to reanalyze the diagnostic morphological characters used to distinguish *B. sulfuratus* from *B. hermogenesi* and redefine their geographical distributions and distributional limits.

## Materials and Methods

The critical analysis of the species identity of specimens attributable to *Brachycephalus hermogenesi*, *B. sulfuratus*, and to a potentially undescribed flea toad from southeastern and southern Brazil provided in our study was based either on the analysis of their morphology or on their advertisement calls. We looked for records in museum specimens, in acoustic collections, and in the literature. The analyzed museum collections include Museu de História Natural Capão da Imbuia (MHNCI), Curitiba, Paraná, Brazil, Coleção Herpetológica do Departamento de Zoologia (DZUP), Universidade Federal do Paraná, Curitiba, Paraná, Brazil, and Museu de História Natural (ZUEC), Universidade Estadual de Campinas, Campinas, São Paulo, Brazil. The sound collection analyzed include MHCNI, Xeno-Canto sound collection (www.xeno-canto.org), and Fonoteca Neotropical Jacques Vielliard (FNJV; https://www2.ib.unicamp.br/fnjv/).

The analyses began by the assessment of the original diagnosis of *B. sulfuratus* (Condez et al., 2016). We looked for the proposed diagnostic characters in museum specimens, calls, sources provided in the literature, and our own photographs of live specimens. Given that this procedure uncovered ambiguity in the proposed diagnostic characters to separate *B. sulfuratus* from *B. hermogenesi*, we sought for new characters that could be useful to distinguish them. New distinctive characters were then erected as diagnostic characters, acting in accordance of the Recommendation 13A of the International Code of Zoological Nomenclature (http://www.iczn.org/).

When comparing the calls between *B. sulfuratus* and *B. hermogenesi*, we noticed that the calls of *B. hermogenesi* described by Verdade et al. (2008) were from a site 112 km distant in a straight line from the type locality of this species (Giaretta & Sawaya, 1998). As this distance is considerable in relation to distances between other species of the genus (Pie et al., 2013; Bornschein et al., 2016a), we made additional recordings in the type localities of *B. hermogenesi* (Núcleo Picinguaba and Corcovado; Giaretta & Sawaya (1998)) and in the locality where Verdade et al. (2008) described the calls of this species (Estação Biológica de Boracéia), as well as in other locations of records of *B. hermogenesi* (e.g., Parque Natural Municipal Nascentes de Paranapiacaba; Verdade, Rodrigues & Pavan, 2009).

Our recordings, deposited in the MHNCI, were made using analogical (Sony TCM–5000EV) and digital (Marantz PMD660, Sony PCM–D50 and PCM–M10 and Tascam DR44-WL) devices, with Sennheiser ME 66 and ME 67 microphones. Analogical recordings were digitized at 44.1 kHz and 16 bit using Raven Pro 1.4 (Cornell Lab of Ornithology, Ithaca, NY, USA). Digital recordings were made equally with sampling frequency rate of 44.1 kHz and 16-bit resolution. We analyzed calls under note-centered approach (Köhler et al., 2017), as Bornschein et al. (2018, 2019) and Pie et al. (2018b). The definition of call used by Condez et al. (2016) is the one defined by Köhler et al. (2017) as note-centered approach, in which several notes emitted continuously over a period represent the call of the species, in contrast to the call-centered approach, in which each note represents a call. Remaining call terminology used were those of Bornschein et al. (2018). Spectrograms were produced using Seewave package, version 2.1.6 (Sueur, Aubin & Simonis, 2008), in R. 4.0.3 (R Core Team, 2018). We made adjustments in contrast and brightness with the intention of lightening the images and best highlighting the pulses. We chose not to noise-filter the spectrograms to avoid eliminating sound characters.

We also included unpublished records in an analysis of *B. sulfuratus* and *B. hermogenesi*, vouchered with specimens collected and deposited in the MHNCI. Collection permits were issued by ICMBIO (10.500, 22470–2/1911426 and 55918–1). Geographical coordinates are based on the WGS84 datum. Elevations for literature records and author’s records were obtained from Google Earth, after plotting the location point (Bornschein et al., 2016a).

Finally, we generated a phylogenetic tree based on a concatenated dataset of all mitochondrial 12S and 16S mitochondrial loci available on GenBank for specimens of the *B. didactylus* species group (Table S1). Sequences were aligned using MAFFT (Katoh et al., 2002) and analyzed under a single GTRGAMMA model in RAxML 8.2.12 (Stamatakis, 2014). Support values were obtained by bootstrapping using the automatic halting option. The final tree was rooted by its midpoint. Whenever possible, the corresponding localities available on their GenBank records were standardized based on the toponyms indicated in Table 1.

**Table 1:** Current identification of records of flea toads at some point identified as *Brachycephalus sulfuratus*, *B. hermogenesi*, and as an unidentified related species, southeastern and southern Brazil.

Species	Locality and state	Geographical coordinates and altitude	Previous identification	Voucher	Our analysis of the record
*B. sulfuratus*					
*B. sulfuratus*	Bairro Rio Vermelho, municipality of Barra do Turvo, São Paulo	24°59′25″S, 48°32′26″W; 790 m a.s.l.	—	Specimen	Specimen examined (MHNCI 11584)
*B. sulfuratus*	Base of the Serra Água Limpa, municipality of Apiaí, São Paulo	24°28′52″S, 48°47′12″W; 920 m a.s.l.	Without species identification: Firkowski et al. (2016); *Brachycephalus* sp. 1: Bornschein et al. (2016a); *B. sulfuratus*: Bornschein et al. (2016b), Ribeiro et al. (2017), Pie et al. (2018b)	Specimen, calls, and genetic sequence on GenBank	Specimen (MHNCI 11583; Fig. 1F) and calls examined (MHNCI 129; Fig. 3B); KX198030.1 analyzed sequence (Fig. 7)
*B. sulfuratus*	Biquinha, municipality of Juquiá, São Paulo	24°17′43″S, 47°36′26″W; 40 m a.s.l.	*B. sulfuratus*: Bornschein, Pie & Teixeira, 2019	Calls	Calls examined (MHNCI 128)
*B. sulfuratus*	Braço do Norte, municipality of Itapoá, Santa Catarina	26°07′29″S, 48°43′48″W; 240 m a.s.l.	*B. sulfuratus*: Monteiro et al. (2018b)	Specimen and genetic sequence on GenBank	MG889430.1 analyzed sequence (Fig. 7)
*B. sulfuratus*	Caratuval, near the Parque Estadual das Lauráceas, municipality of Adrianópolis, Paraná	24°51′17″S, 48°43′43″W; 900 m a.s.l.	Without species identification: Firkowski et al. (2016); *Brachycephalus* sp. nov. 1: Pie et al. (2013); *Brachycephalus* sp. 1: Bornschein et al. (2016a); *B. sulfuratus*: Bornschein et al. (2016b), Ribeiro et al. (2017), Pie et al. (2018b)	Specimen, calls, and genetic sequence on GenBank	Specimen (MHNCI 11571; Fig. 1B) and calls examined (MHNCI 131); KX198031.1 analyzed sequence (Fig. 7)
*B. sulfuratus*	Caratuval, Parque Estadual das Lauráceas, municipality of Adrianópolis, Paraná	24°51′14″S, 48°42′01″W; 890 m a.s.l.	*Brachycephalus* sp. nov. 1: Pie et al. (2013); *Brachycephalus* sp. 1: Bornschein et al. (2016a)	Calls	Calls examined (MHNCI 132)
*B. sulfuratus*	Castelo dos Bugres, municipality of Joinville, Paraná	26°13′47″S, 49°03′20″W; 790–860 m a.s.l.	*Brachycephalus* sp. nov. 1: Pie et al. (2013); *Brachycephalus* sp. 1: Bornschein et al. (2016a); *B. sulfuratus*: Condez et al. (2016), Monteiro et al. (2018b)	Specimen, calls, and genetic sequence on GenBank	MK697439.1, MK697487.1, KU321533.1, and MK697390.1 analyzed sequence (Fig. 7)
*B. sulfuratus*	Centro de Estudos e Pesquisas Ambientais da Univille, Vila da Glória, Distrito do Saí, municipality of São Francisco do Sul, Santa Catarina	26°13′39″S, 48°41′31″W; 125 m a.s.l.	*B. sulfuratus*: Condez et al. (2016)	Specimen, calls, and genetics	—
*B. sulfuratus*	Corvo, municipality of Quatro Barras, Paraná	25°20′17″S, 48°54′56″W; 930 m a.s.l.	Without species identification: Firkowski et al. (2016); *Brachycephalus* sp. nov. 1: Pie et al. (2013); *Brachycephalus* sp. 1: Bornschein et al. (2016a); *B. sulfuratus*: Bornschein et al. (2016b), Ribeiro et al. (2017), Pie et al. (2018b, 2018a)	Specimen and genetic sequence on GenBank	Specimen examined (MHNCI 10788, MHNCI 11573, MHNCI 11575; Figs. 1A, 1E, and 1I); KX198033.1 analyzed sequence (Fig. 7)
*B. sulfuratus*	Entroncamento Teba, Rio Turvo, municipality of Campina Grande do Sul, Paraná	25°01′28″S, 48°37′12″W; 785 m a.s.l.	—	Specimens and calls	Specimens (MHNCI 11586–7) and calls examined (MHNCI 219)
*B. sulfuratus*	Estância Hidroclimática Recreio da Serra, Serra da Baitaca, municipality of Piraquara, Paraná	25°27′14″S, 49°00′28″W; 1,150–1,205 m a.s.l.	*B. sulfuratus*: Bornschein, Pie & Teixeira, 2019	Specimen	Specimen examined (MHNCI 11591)
*B. sulfuratus*	Fazenda Thalia, municipality of Balsa Nova, Paraná	25°30′58″S, 49°40′12″W; 1,025 m a.s.l.	Without species identification: Firkowski et al. (2016); *Brachycephalus* sp. nov. 1: Pie et al. (2013); *Brachycephalus* sp. 1: Bornschein et al. (2016a); *B. sulfuratus*: Bornschein et al. (2016b), Ribeiro et al. (2017), Pie et al. (2018b)	Specimens, calls, and genetic sequence on GenBank	Specimens (MHNCI 11579–81, MHNCI 11582; Figs. 1C, 1D, 1G and 1H ) and calls examined (MHNCI 134); KX198032.1 analyzed sequence (Fig. 7)
*B. sulfuratus*	near the Jurupará dam, municipality of Piedade, São Paulo	23°56′30″S, 47°23′45″W; 690 m a.s.l.	*B. sulfuratus*: Pie et al. (2018b)	Specimens and calls	Specimens (MHNCI 10790–2; Figs. 1J and 1L ) and calls examined (MHNCI 123–5; Figs. 3A, 3C and 3D)
*B. sulfuratus*	Mananciais da Serra, municipality of Piraquara, Paraná	25°29′32″S, 48°59′33″W; 970–1,050 m a.s.l.	*Brachycephalus* sp. nov. 1: Pie et al. (2013); *Brachycephalus* sp. 1: Bornschein et al. (2016a); *B. sulfuratus*: Bornschein et al. (2016b), Ribeiro et al. (2017), Pie et al. (2018b)	Specimen	Specimen examined (MHNCI 10302)
*B. sulfuratus*	Monte Crista, municipality of Garuva, Santa Catarina	26°04′53″S; 48°55′03″W; 435 m a.s.l.	—	Calls	Calls examined (MHNCI 221)
*B. sulfuratus*	Morro Anhangava, municipality of Quatro Barras, Paraná	25°22′51″S, 49°01′26″W; 915 m a.s.l.	*B. sulfuratus*: Condez et al. (2016), Monteiro et al. (2018b)	Specimen and genetic sequence on GenBank	MK697488.1, MK697440.1, KU321534.1, and MG889428.1 analyzed sequences (Fig. 7)
*B. sulfuratus*	Morro do Canal, municipality of Piraquara, Paraná	25°30′55″S; 48°58′56″W; 1,315 m	—	Calls	Calls examined (MHNCI 220)
*B. sulfuratus*	Morro do Cantagalo, Vila da Glória, Distrito do Saí, municipality of São Francisco do Sul, Santa Catarina	26°10′31″S, 48°42′44″W; 160 m a.s.l.	*B. sulfuratus*: Condez et al. (2016)	Specimen and genetic sequence on GenBank	MK697441.1, MK697489.1, KU321532.1, and MK697392.1 analyzed sequences (Fig. 7)
*B. sulfuratus*	Morro do Garrafão, municipality of Corupá, Santa Catarina	26°28′23″S, 49°15′57″W; 500–530 m a.s.l.	*B. sulfuratus*: Pie et al. (2018b), Teixeira et al. (2018)	Specimen and calls	Specimens (MHNCI 10826-8; Fig. 1K) and calls examined (MHNCI 137)
*B. sulfuratus*	Morro Garuva, municipality of Garuva, Santa Catarina	26°02′29″S, 48°53′14″W; 215–495 m a.s.l.	*B. sulfuratus*: Bornschein, Pie & Teixeira, 2019	Calls	Calls examined (MHNCI 136)
*B. sulfuratus*	Municipality of Barra do Turvo	c. 24°45′S, 48°29′W; altitude?	*B. sulfuratus*: GenBank	Genetic sequence on GenBank	MK697486.1, MK697438.1, and MK697389.1 analyzed sequences (Fig. 7)
*B. sulfuratus*	Municipality of Piedade, São Paulo	c. 23°54′S, 47°25′W; altitude?	*B. hermogenesi*: Condez, Sawaya & Dixo (2009), Clemente-Carvalho et al. (2011); *Brachycephalus* sp. cf. *B. sulfuratus* or *B. hermogenesi*: Bornschein, Pie & Teixeira, 2019	Specimen and genetic sequence on GenBank	HQ435682.1 and HQ435709.1 analyzed sequences (Fig. 7)
*B. sulfuratus*	Núcleo Itutinga-Pilões, Parque Estadual da Serra do Mar, municipality of Cubatão, São Paulo	23°54′17″S, 46°29′22″W; 55 m a.s.l.	*B. sulfuratus*: Bornschein, Pie & Teixeira, 2019	Calls	Calls examined (MHNCI 126–7)
*B. sulfuratus*	Parque Estadual da Ilha do Cardoso, municipality of Cananéia, São Paulo	25°06′53″S, 47°55′40″W; 385 m a.s.l.	Possibly *B. hermogenesi*: Verdade et al. (2008); *B. sulfuratus*: Condez et al. (2016)	Specimen, calls, and genetic sequence on GenBank	MK697485.1, MK697437.1, KU321535.1, and MK697388.1 analyzed sequences (Fig. 7)
*B. sulfuratus*	Parque Estadual Intervales, municipality of Iporanga, São Paulo	24°16′33″S, 48°25′04″W; 820 m a.s.l.	*B. sulfuratus*: Bornschein, Pie & Teixeira, 2019	Calls	Calls examined (XC80463 XC18179, XC75544)
*B. sulfuratus*	Pedra da Tartaruga, municipality of Garuva, Santa Catarina	25°59′42″S, 48°54′23″W; 465 m a.s.l.	—	Specimen	Specimen examined (MHNCI 11585)
*B. sulfuratus*	Pico Marumbi, Parque Estadual do Pico Marumbi, municipality of Morretes, Paraná	25°27′03″S; 48°54′59″W; 1180 m a.s.l.	—	Specimen	Specimen examined (MHNCI 10302)
*B. sulfuratus*	Recanto das Hortências, municipality of São José dos Pinhais, Paraná	25°33′24″S, 48°59′38″W; 975 m a.s.l.	*Brachycephalus* sp. 1: Bornschein et al. (2016a); *B. sulfuratus*: Ribeiro et al. (2017), Bornschein et al. (2016b), Pie et al. (2018b)	Specimen	Specimen examined
*B. sulfuratus*	Reserva Particular do Patrimônio Natural Salto Morato, municipality of Guaraqueçaba, Paraná	25°09′14″S, 48°18′06″W; 40–880 m a.s.l.	*B. hermogenesi*: Pereira et al. (2010), Santos-Pereira et al. (2011, 2016), Santos-Pereira, Pombal & Rocha (2018), Leivas et al. (2018); *Brachycephalus* sp. 1: Bornschein et al. (2016a)	Specimen and calls	Calls examined (MHNCI 133)
*B. sulfuratus*	Salto do Inferno, Rio Capivari, municipality of Bocaiúva do Sul, Paraná	25°00′02″S, 48°37′07″W; 610 m a.s.l.	*B. sulfuratus*: Ribeiro et al. (2017), Bornschein et al. (2016b), Pie et al. (2018b)	Specimen	Specimen examined
*B. sulfuratus*	Serra do Guaraú, on the border of the municipalities of Cajati and Jacupiranga, São Paulo	24°47′12″S, 48°07′11″W; 680–835 m a.s.l.	*B. sulfuratus*: Bornschein, Pie & Teixeira, 2019	Calls	Calls examined (MHNCI 130)
*B. sulfuratus*	Serra do Pico, municipality of Joinville, Santa Catarina	26°08′31″S, 48°57′19″W; 340–720 m a.s.l.	*B. sulfuratus*: Bornschein, Pie & Teixeira, 2019	Calls	Calls examined (MHNCI 217)
*B. sulfuratus*	Torre Embratel, municipality of Cajati, São Paulo	24°52′46″S, 48°15′27″W; 960–990 m a.s.l.	*B. sulfuratus*: Bornschein, Pie & Teixeira, 2019	Specimen and calls	Specimen (MHNCI 11588) and calls examined (MHNCI 218)
*B. sulfuratus*	Truticultura, municipality of Garuva, Paraná	26°01′33″S, 48°52′02″W; 90 m a.s.l.	*Brachycephalus* sp. nov. 1: Pie et al. (2013); *Brachycephalus* sp. 1: Bornschein et al. (2016a); *B. sulfuratus*: Bornschein, Pie & Teixeira, 2019	Calls	Calls examined (MHNCI 135)
*B. hermogenesi*					
*B. hermogenesi*	Corcovado, municipality of Ubatuba, São Paulo	23°28′20″S, 45°11′41″W; 30–250 m a.s.l.	*B. hermogenesi*: Bornschein, Pie & Teixeira, 2019 ; in *part*.)	Calls	Calls examined (MHNCI 166; Figs. 4A and 4D)
*B. hermogenesi*	Estação Biológica de Boracéia, municipality of Salesópolis, São Paulo	23°39′10″S, 45°53′05″W; 825–900 m a.s.l.	*B. hermogenesi*: Pimenta, Bérnils & Pombal (2007), Verdade et al. (2008), Pie et al. (2013), Bornschein et al. (2016a), Condez et al. (2016)	Specimens and calls	Specimens (MHNCI, one uncatalogued specimen) and calls examined (MHNCI 166-9; Fig. 4E), including recordings sent by V. K. Verdade
*B. hermogenesi*	Fazenda Capricórnio, municipality of Ubatuba, São Paulo	23°23′27″S, 45°04′26″W; 60 m a.s.l.	*B. hermogenesi*: Giaretta & Sawaya (1998), Verdade et al. (2008), Pie et al. (2013), Bornschein et al. (2016a), Condez et al. (2016)	Specimens (paratypes)	Specimen examined (ZUEC 9725)
*B. hermogenesi*	Morro do Cantagalo, municipality of Caraguatatuba, São Paulo	23°36′23″S, 45°23′34″W; 155-195 m a.s.l.	—	Calls	Calls examined (MHNCI 222-3)
*B. hermogenesi*	Municipality of Paraibuna, São Paulo	c. 23°23′34″S, 45°39′42″W; altitude?	*B. hermogenesi*: Condez et al. (2016)	Specimen and genetic sequence on GenBank	MK697373.1 analyzed sequence (Fig. 7)
*B. hermogenesi*	Núcleo Cunha, Parque Estadual da Serra do Mar, municipality of Cunha, São Paulo	23°15′48″S, 45°02′39″W; 1,045–1,140 m a.s.l.	*B. hermogenesi*: Bornschein, Pie & Teixeira, 2019	Specimen and calls	Specimen (MHNCI, one uncatalogued specimen) and calls examined (MHNCI 170-1)
*B. hermogenesi*	Núcleo Picinguaba, Parque Estadual da Serra do Mar, municipality of Ubatuba, São Paulo	23°22′21″S, 44°49′53″W; 0–700 m a.s.l.	*B. hermogenesi*: Giaretta & Sawaya (1998), Pimenta, Bérnils & Pombal (2007), Verdade et al. (2008), Clemente-Carvalho et al. (2009), Pie et al. (2013), Bornschein et al. (2016a), Condez et al. (2016), Pie et al. (2018b)	Specimens (holotype and paratypes), calls, and genetic sequence on GenBank	Specimens (ZUEC 9715–21; Fig. 3D) and calls examined (MHNCI 172-87; Figs. 4B, 4C and 4F); MK697472.1, KU321531.1, and MK697374.1 analyzed sequences (Fig. 7)
*B. hermogenesi*	Núcleo Santa Virgínea, Parque Estadual da Serra do Mar, municipality of São Luiz do Paraitinga, São Paulo	23°19′36″S, 45°07′57″W; 915 m a.s.l.	—	Calls	Calls examined (XC253045)
*B. hermogenesi*	Parque Natural Municipal Nascentes de Paranapiacaba, municipality of Santo André, São Paulo	23°46′10″S, 46°17′36″W; 840 m a.s.l.	*B. hermogenesi*: Verdade, Rodrigues & Pavan (2009); *Brachycephalus* sp. cf. *B. sulfuratus* or *B. hermogenesi*: Bornschein, Pie & Teixeira, 2019	Calls	Calls examined (MHNCI 213-6)
*B. hermogenesi*	Sertão da Cutia, municipality of Ubatuba, So Paulo	not located	*B. hermogenesi*: Condez et al. (2016)	Specimen	—
*B. hermogenesi*	Trilha do Ipiranga 50 m from the Rio Ipiranga, Núcleo Santa Virgínia, Parque Estadual da Serra do Mar, municipality of São Luiz do Paraitinga, São Paulo	23°20′41″S, 45°08′21″W; 920–940 m a.s.l.	*B. hermogenesi*: Bornschein, Pie & Teixeira, 2019	Calls	Calls examined (MHNCI 188-92)
*Brachycephalus* sp. (other than *B. sulfuratus* and *B. hermogenesi*)					
*Brachycephalus* sp.	Corcovado, municipality of Ubatuba, São Paulo	23°28′20″S, 45°11′41″W; 30–250 m a.s.l.	*B. hermogenesi*: Giaretta & Sawaya (1998), Verdade et al. (2008), Pie et al. (2013), Bornschein et al. (2016a), Pie et al. (2018b); collected at “Picinguaba” [= Corcovado]), Bornschein, Pie & Teixeira, 2019 in *part*.)	Specimens (including paratypes) and calls	Specimens (ZUEC 9722-4, MHNCI 10823-5) and calls examined (MHNCI 193–205; Figs. 5A–5C)
*Brachycephalus* sp.	Trilha do Corisco, municipality of Paraty, Rio de Janeiro	23°16′38″S, 44°46′39″W; 350–725 m a.s.l.	*B. hermogenesi*: Bornschein, Pie & Teixeira, 2019	Calls	Calls examined (MHNCI 206–12; Fig. 5D)
*Brachycephalus* sp. (*B. hermogenesi* or *B. sulfuratus*)					
*Brachycephalus* sp.	Alto Quiriri, municipality of Garuva, Santa Catarina	26°05′34″S, 48°59′41″W; 240 m a.s.l.	*Brachycephalus* sp. nov. 1: Pie et al. (2013); *Brachycephalus* sp. 1: Bornschein et al. (2016a); *Brachycephalus* sp. cf. *B. sulfuratus*: Bornschein, Pie & Teixeira, 2019	Unvouchered	The calls resemble those of *B. sulfuratus* (auditory record made by MRB)
*Brachycephalus* sp.	Colônia Castelhanos, municipality of Guaratuba, Paraná	25°47′58″S, 48°54′40″W; 290 m a.s.l.	*Brachycephalus* aff. *hermogenesi*: Cunha, Oliveira & Hartmann (2010); *B. hermogenesi* Oliveira et al. (2011); *Brachycephalus* sp. nov. 1: Pie et al. (2013); *Brachycephalus* sp. 1: Bornschein et al. (2016a); *B. sulfuratus*: Condez et al. (2016); *Brachycephalus* sp. cf. *B. sulfuratus*: Bornschein, Pie & Teixeira, 2019	Specimen	Specimen examined (ZUEC 16602)
*Brachycephalus* sp.	Dona Francisca, municipality of Joinville, Santa Catarina	26°09′52″S, 48°59′23″W; 150 m a.s.l.	*Brachycephalus* sp. nov. 1: Pie et al. (2013); *Brachycephalus* sp. 1: Bornschein et al. (2016a); *Brachycephalus* sp. cf. *B. sulfuratus*: Bornschein, Pie & Teixeira, 2019	Unvouchered	The calls resemble those of *B. sulfuratus* (auditory record made by MRB)
*Brachycephalus* sp.	Estação Ecológica Juréia-Itatins, municipality of Iguape, São Paulo	c. 24°27′S, 47°24′W; altitude?	*B. hermogenesi*: Verdade et al. (2008); *Brachycephalus* sp. cf. *B. sulfuratus* or *B. hermogenesi*: Bornschein, Pie & Teixeira, 2019	Specimen	—
*Brachycephalus* sp.	Estrada do Rio do Júlio, municipality of Joinville, Santa Catarina	26°17′02″S, 49°06′08″W; 650 m a.s.l.	*Brachycephalus* sp.: Mariotto (2014); *Brachycephalus* sp. 1: Bornschein et al. (2016a); *Brachycephalus* sp. cf. *B. sulfuratus*: Bornschein, Pie & Teixeira, 2019	Specimen	—
*Brachycephalus* sp.	Fazenda Pico Paraná, municipality of Campina Grande do Sul, Paraná	25°13′29″S, 48°51′17″W; 1,050–1,085 m a.s.l.	*Brachycephalus* sp. nov. 1: Pie et al. (2013); *Brachycephalus* sp. 1: Bornschein et al. (2016a); *Brachycephalus* sp. cf. *B. sulfuratus*: Bornschein, Pie & Teixeira, 2019	Unvouchered	The calls resemble those of *B. sulfuratus* (auditory records made by MRB and LFR)
*Brachycephalus* sp.	Fazenda Primavera, municipality of Tunas do Paraná, Paraná	24°53′08″S, 48°45′51″W; 1,060 m a.s.l.	*Brachycephalus* sp. nov. 1: Pie et al. (2013); *Brachycephalus* sp. 1: Bornschein et al. (2016a); *Brachycephalus* sp. cf. *B. sulfuratus*: Bornschein, Pie & Teixeira, 2019	Unvouchered	The calls resemble those of *B. sulfuratus* (auditory record made by MRB)
*Brachycephalus* sp.	Municipality of Ibiúna, São Paulo	c. 23°39′S, 47°13′W; altitude?	*B. hermogenesi*: Condez et al. (2016); *Brachycephalus* sp. cf. *B. sulfuratus* or *B. hermogenesi*: Bornschein, Pie & Teixeira, 2019	Specimen	—
*Brachycephalus* sp.	Municipality of Juquitiba, São Paulo	c. 23°56′S, 47°04′W; altitude?	*B. hermogenesi*: Verdade et al. (2008), Condez et al. (2016); *Brachycephalus* sp. cf. *B. sulfuratus* or *B. hermogenesi*: Bornschein, Pie & Teixeira, 2019	Specimen	—
*Brachycephalus* sp.	Municipality of Peruíbe, São Paulo	24°18′S, 46°59′W; altitude?	*B. hermogenesi*: Condez et al. (2016); *Brachycephalus* sp. cf. *B. sulfuratus* or *B. hermogenesi*: Bornschein, Pie & Teixeira, 2019	Specimen	—
*Brachycephalus* sp.	Municipality of Registro, São Paulo	c. 24°30′S, 47°51′W; altitude?	*B. hermogenesi*: Condez et al. (2016); *Brachycephalus* sp. cf. *B. sulfuratus* or *B. hermogenesi*: Bornschein, Pie & Teixeira, 2019	Specimen	—
*Brachycephalus* sp.	Municipality of Ribeirão Grande, São Paulo	c. 24°06′S, 48°22′W; altitude?	*B. hermogenesi*: Verdade et al. (2008); *Brachycephalus* sp. cf. *B. sulfuratus* or *B. hermogenesi*: Bornschein, Pie & Teixeira, 2019	Specimen	—
*Brachycephalus* sp.	Municipality of Tapiraí, São Paulo	c. 23°57′55″S, 47°30′19″W; 870 m a.s.l.	*B. hermogenesi*: Verdade et al. (2008), Condez, Sawaya & Dixo (2009); *Brachycephalus* sp. cf. *B. sulfuratus* or *B. hermogenesi*: Bornschein, Pie & Teixeira, 2019	Specimen	—
*Brachycephalus* sp.	Parque Estadual de Jacupiranga, municipality of Eldorado, São Paulo	c. 24°38′S, 48°24′W; altitude?	*B. hermogenesi*: Condez et al. (2016); *Brachycephalus* sp. cf. *B. sulfuratus* or *B. hermogenesi*: Bornschein, Pie & Teixeira, 2019	Specimen	—
*Brachycephalus* sp.	Pico Agudinho, Serra da Prata, municipality of Morretes, Paraná	25°36′24″S, 48°43′33″W; 385 m a.s.l.	*Brachycephalus* sp. nov. 1: Pie et al. (2013); *Brachycephalus* sp. 1: Bornschein et al. (2016a); *Brachycephalus* sp. cf. *B. sulfuratus*: Bornschein, Pie & Teixeira, 2019	Unvouchered	The calls resemble those of *B. sulfuratus* (auditory record made by MRB)
*Brachycephalus* sp.	Reserva Betary, municipality of Iporanga, São Paulo	24°33′08″S, 48°40′49″W; 190 m a.s.l.	*Brachycephalus* sp. cf. *B. sulfuratus* or *B. hermogenesi*: Bornschein, Pie & Teixeira, 2019	Specimen	Specimen examined (ZUEC 19931)
*Brachycephalus* sp.	Reserva Biológica do Alto da Serra de Paranapiacaba, municipality of Santo André, São Paulo	23°46′40″S, 46°18′45″W; 800–850 m a.s.l.	*B. hermogenesi*: Verdade et al. (2008), Verdade, Rodrigues & Pavan (2009); *Brachycephalus* sp. cf. *B. sulfuratus* or *B. hermogenesi*: Bornschein, Pie & Teixeira, 2019	Unvouchered	—
*Brachycephalus* sp.	Reserva Florestal de Morro Grande, municipality of Cotia, São Paulo	23°42′08″S, 46°58′22″W; cf. 990 m a.s.l.	*B. hermogenesi*: Dixo & Verdade (2006), Verdade et al. (2008), Condez et al. (2016); *Brachycephalus* sp. cf. *B. sulfuratus* or *B. hermogenesi*: Bornschein, Pie & Teixeira, 2019	Specimen	—
*Brachycephalus* sp.	Sítio Ananias, municipality of Guaratuba, Paraná	25°47′08″S, 48°43′03″W; 25 m a.s.l.	*Brachycephalus* sp. nov. 1: Pie et al. (2013); *Brachycephalus* sp. 1: Bornschein et al. (2016a); *Brachycephalus* sp. cf. *B. sulfuratus*: Bornschein, Pie & Teixeira, 2019	Unvouchered	The calls resemble those of *B. sulfuratus* (auditory record made by MRB)
*Brachycephalus* sp. (*B. hermogenesi* or *Brachycephalus* sp. from Corcovado and Trilha do Corisco)					
*Brachycephalus* sp.	Morro Cuscuzeiro, on the border of municipalities of Paraty, Rio de Janeiro, and Ubatuba, São Paulo	23°17′50″S, 44°47′21″W; 730–1,090 a.s.l.	*B. hermogenesi*: Bornschein, Pie & Teixeira, 2019	Unvouchered	The calls resemble those of *Brachycephalus* sp. of Trilha do Corisco (auditory record made by MRB and LFR)
*Brachycephalus* sp.	Morro do Corcovado, Parque Estadual da Serra do Mar, municipality of Ubatuba, São Paulo	23°27′06″S, 45°12′03″W; 250–1,060 m a.s.l.	*B. hermogenesi*: Bornschein, Pie & Teixeira, 2019	Unvouchered	The calls resemble those of *Brachycephalus* sp. of Trilha do Corisco (auditory record made by MRB and LFR)
*Brachycephalus* sp	Municipality of Paraty, Rio de Janeiro	c. 23°13′07″S, 44°43′15″W; altitude?	*B. hermogenesi*: Giaretta & Sawaya (1998); *Brachycephalus* sp. cf. *B. hermogenesi*: Bornschein, Pie & Teixeira, 2019	Unvouchered	—

**Note:**

Our revision resulted in some unidentified records (*B. sulfuratus*, *B. hermogenesi* or a third species); the probable identifications are provided below. Localities are in alphabetical order (accordingly to the respective species). Abbreviations: FNJV = fonoteca neotropical Jacques Vielliard; MHNCI = Museu de História Natural Capão da Imbuia, Curitiba, Paraná, Brazil; ZUEC = Museu de História Natural, Universidade Estadual de Campinas, Campinas, state of São Paulo, Brazil; XC = Xeno-Canto sound collection (www.xeno-canto.org).

## Results

Our list of specimens and calls analyzed of *B. sulfuratus* and *B. hermogenesi*, per locality, is provided in Table 1 and Appendix 1.

### Diagnosis between *Brachycephalus sulfuratus* and *B. hermogenesi*

**Figure 1:**
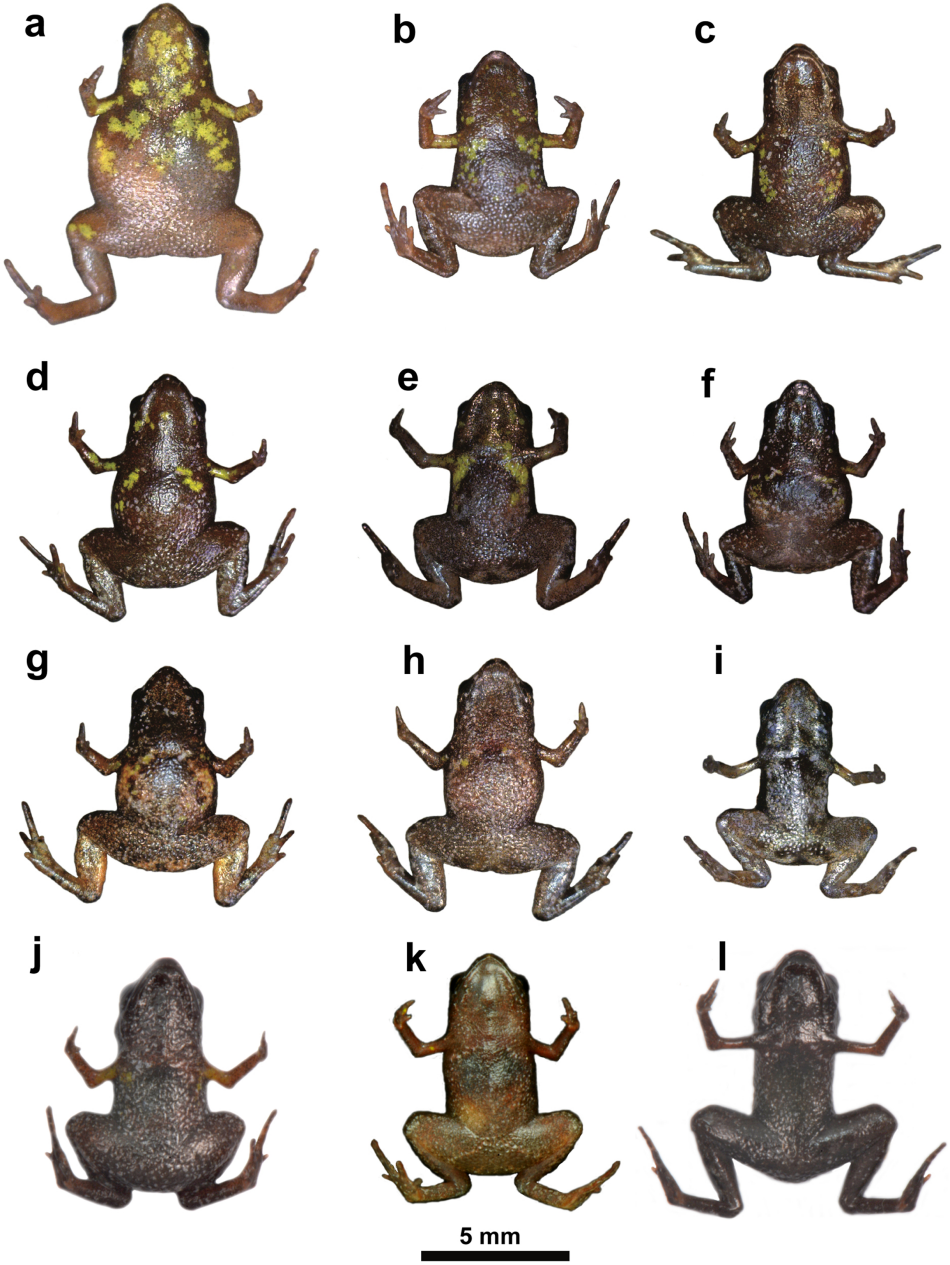
Ventral view of live specimens of *Brachycephalus sulfuratus*. Ventral view of live specimens of *Brachycephalus sulfuratus* initially deposited in DZUP) and transferred to MHNCI. (A) MHNCI 11575 (ex-DZUP 153) (Corvo, Paraná); (B) MHNCI 11571 (ex-DZUP 139)(Caratuval, near the Parque Estadual das Lauráceas, Paraná); (C) MHNCI 11582 (ex-DZUP 224) (Fazenda Thalia, Paraná); (D) MHNCI 11579 (ex-DZUP 221) (Fazenda Thalia); (E) MHNCI 11573 (ex-DZUP 151) (Corvo); (F) MHNCI 11583 (ex-DZUP 362) (base of the Serra Água Limpa, São Paulo); (G) MHNCI 11580 (ex-DZUP 222) (Fazenda Thalia); (H) MHNCI 11581 (ex-DZUP 223) (Fazenda Thalia); (I) MHNCI 10788 (ex-DZUP 154) (Corvo); (J) MHNCI 10790 (near the Jurupará dam, São Paulo); (K) MHNCI 10826 (Morro do Garrafão, Santa Catarina); (L) MHNCI 10792 (near the Jurupará dam). Notice the variable of yellow spots, absent in specimen “l”, as well as the absence of the dark-brown inverted v-shaped mark on the cloacal region of specimen “a”. Compare sonograms from specimens “j” and “l” in Figs. 2B and 2C. The presence of yellow spots and v-shaped mark was proposed as diagnostic characteristics to distinguish *B. sulfuratus* from *B. hermogenesi*, but they are variable intraspecifically. For details on geographical localities, see Table 1. Photo credit: Luiz Fernando Ribeiro.

Condez et al. (2016) indicated three morphological characters to diagnose *B. sulfuratus* from the very similar *B. hermogenesi*: (1) It “differs from*… B. hermogenesi*… by having (in life) yellow blotches on the ventral surfaces of the throat, chest, arms, and forearms” (Condez et al., 2016: 43, 50); (2) a more evident “singular inverted v-shaped mark around the cloacal region in ventral view”, that is “generally rounded and not ornamented in… *B. hermogenesi*…” (Condez et al., 2016: 43, 50); and (3) the presence of an “m-shaped mark around the cloacal opening [in dorsal view], which is… not clearly defined in *B. hermogenesi*” (Condez et al., 2016: 50). Specimens of *B. sulfuratus* collected in southern São Paulo, Paraná and Santa Catarina (Table 1) have revealed that the yellow spots on the ventral surface of this species might still be present, on the throat, chest, arms, and/or forearms, but not necessarily in all of these body parts. In addition, the amount of yellow is highly variable, being virtually absent in some individuals (Fig. 1). Moreover, in three individuals of *B. sulfuratus* collected by us in the state of São Paulo (near the Jurupará dam; Table 1), two do not present yellow spots on the ventral surface (see one of them in Fig. 1L), being identified as *B. sulfuratus* by their advertisement calls (MHNCI 123–5; see below). The inverted v-shaped mark can be absent in individuals of *B. sulfuratus* (compare Fig. 6A of Condez et al. (2016) and Fig. 1A). Additionally, the use of this character is inconsistent as a diagnosis from *B. hermogenesi* on the actual original description: “the ventral inverted v-shaped mark… are shared among the four species (*B. sulfuratus*, *B. hermogenesi*, *B. didactylus* and *B. pulex*)” (Condez et al., 2016: 50). Also, while describing the variation on the type series, the authors stated that “some individuals present the inverted v-shaped around the cloacal region” (Condez et al., 2016: 46). Finally, the “m-shaped mark around the cloacal opening” was also mischaracterized as a diagnostic character on the actual original description of the species (Condez et al., 2016: 50): “The m-shaped mark… are shared among the four species (*B. sulfuratus*, *B. hermogenesi*, *B. didactylus*, and *B. pulex*).”

**Figure 2:**
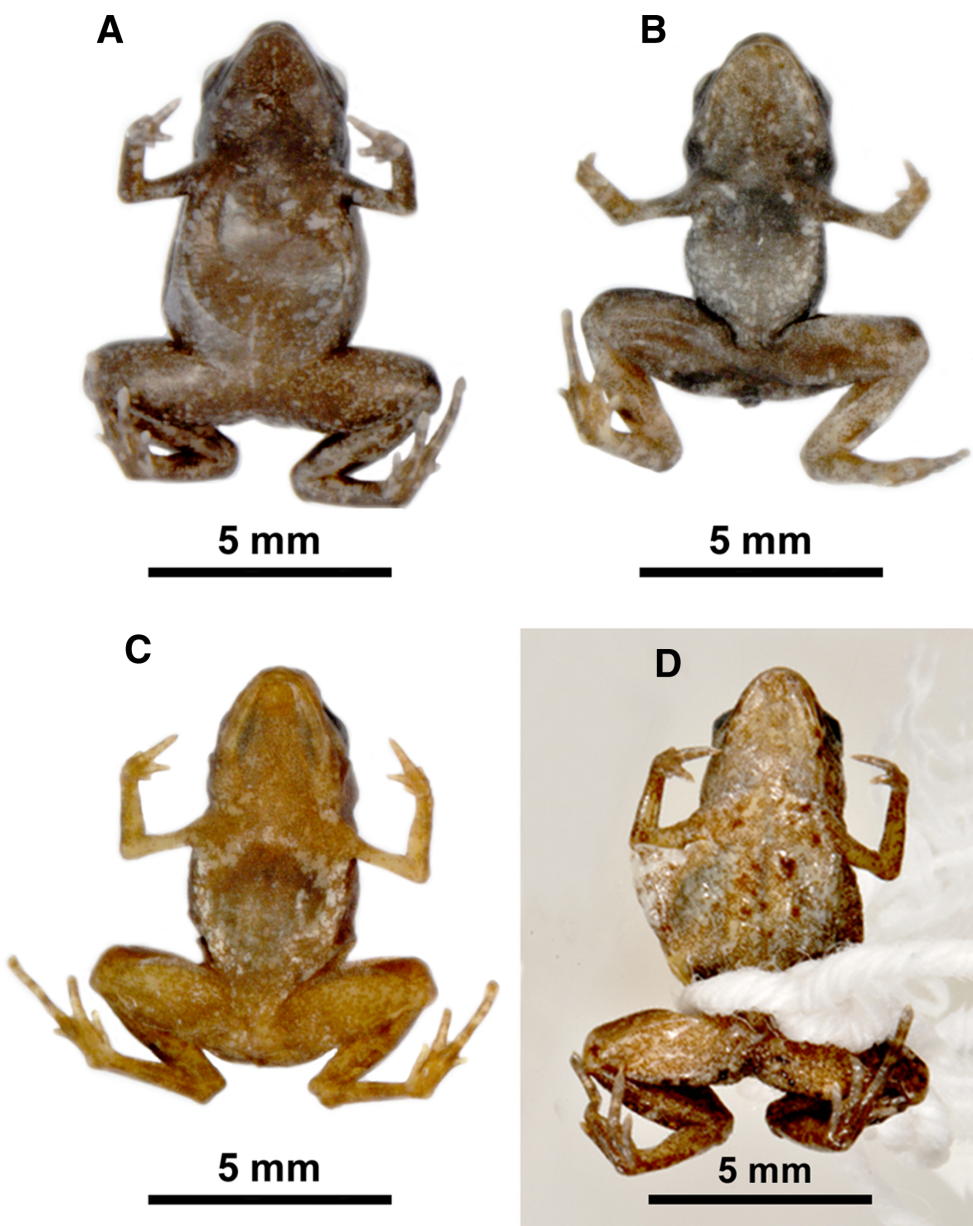
Ventral view of specimens of *Brachycephalus sulfuratus* and *B. hermogenesi*. Ventral view of specimens of *Brachycephalus sulfuratus* (A–C) and *B. hermogenesi* (D) in preservative, deposited in MHNCI and ZUEC: (A) MHNCI 9800 (Salto do Inferno, Paraná); (B) MHNCI 10302 (Mananciais da Serra, Paraná); (C) MHNCI 10303 (Corvo, Paraná; ex DZUP 589); and (D) ZUEC 9715 (Núcleo Picinguaba, São Paulo; holotype of *B. hermogenesi*). Notice the variation in ventral coloration. For details on geographical localities, see Table 1. Photo credit: Luiz Fernando Ribeiro.

Currently, there are no unique morphological character that could differentiate either live or preserved specimens (Fig. 2) for *B. sulfuratus* from *B. hermogenesi*. However, for identification purposes, we considered individuals with yellow spots on their ventral side as *B. sulfuratus*, whereas individuals without yellow spots could be either *B. sulfuratus* or *B. hermogenesi*. It is important to note that specimens with yellow spots of *B. sulfuratus* must be observed in life because in the preservative the change in color prevents separate them in relation to specimens of *B. hermogenesi*.

In addition to morphological characters, Condez et al. (2016: 43) included in the diagnosis of *B. sulfuratus* the following parameters of the advertisement call: “advertisement call long, composed of a set of 4–7 high-frequency notes (6.2–7.2 kHz) repeated regularly.” In the section “Comparisons with other species”, Condez et al. (2016: 50) stating that “The advertisement call of *B. hermogenesi* is the most similar to the new species (*B. sulfuratus*), being quite similar in frequency (dominant frequency = 6.8 kHz), which are the highest recorded for the genus. However, the advertisement call of *B. hermogenesi* can be simple or composed of 2–7 shorter notes with 1–3 pulses (Verdade et al., 2008).” In summary, the indicated values overlap with those of *B. hermogenesi*. The advertisement call of *B. hermogenesi* is composed of 1–7 notes, whereas that of *B. sulfuratus* is composed of 4–7 notes and the amplitude of the dominant frequency of *B. hermogenesi* (6.8 kHz) is within the range of *B. sulfuratus* (6.2–7.2).

**Figure 3:**
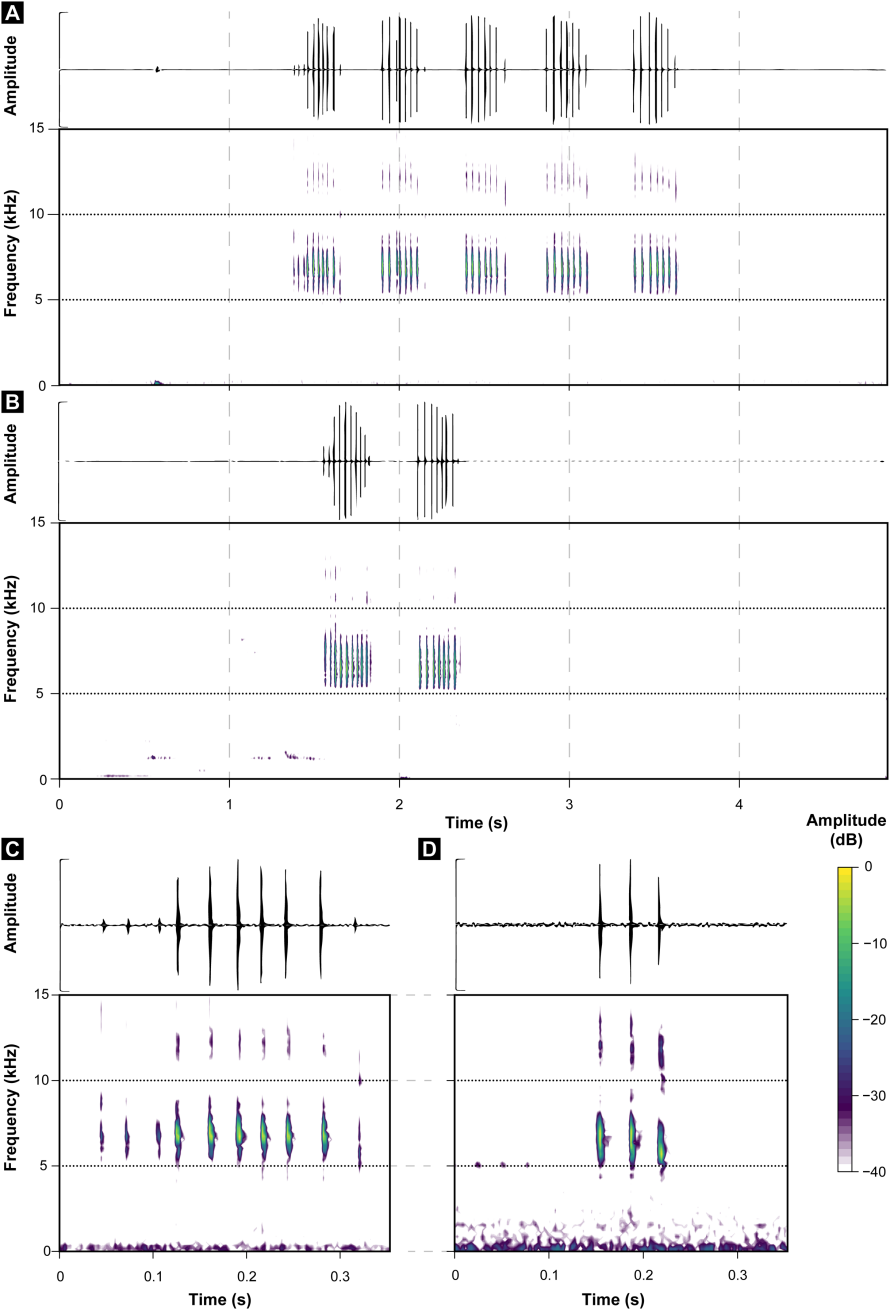
Oscillograms and spectrograms of *Brachycephalus sulfuratus*. (A) Example of one entire call with five notes (MHNCI 124; voucher MHNCI 10791 or MHNCI 10792; near the Jurupará dam, municipality of Piedade, São Paulo; M. R. Bornschein). (B) Example of one entire call with two notes (MHNCI 129; voucher MHNCI 11583; Base of the Serra Água Limpa, municipality of Apiaí, São Paulo; M. R. Bornschein). (C) Example of one note with 10 pulses (MHNCI 124). (D) Example of one note with three pulses (MHNCI 124). Spectrograms are produced with Hann window, overlap of 50%, and FFT size of 512 points in A and B and 256 points in (C) and (D). For details on geographical localities, see Table 1.

**Figure 4:**
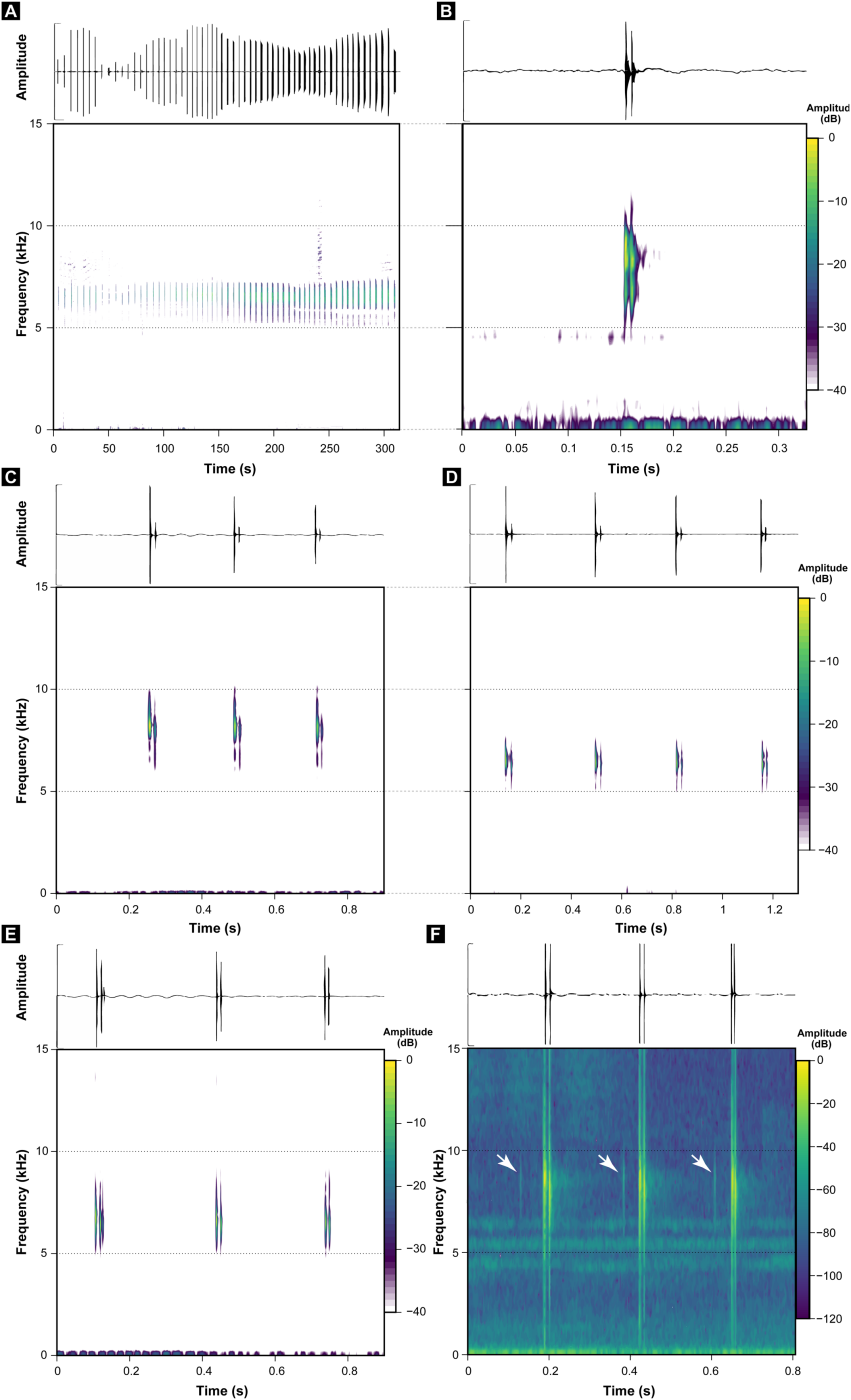
Oscillograms and spectrograms of *Brachycephalus hermogenesi*. (A) Example of one entire call with 135 notes recorded (MHNCI 165; Corcovado, municipality of Ubatuba, São Paulo; L. F. Ribeiro). (B) Example of one isolated note with two pulses (MHNCI 183; Núcleo Picinguaba, Parque Estadual da Serra do Mar, municipality of Ubatuba, São Paulo ; M. R. Bornschein). (C) Example of one note group with three notes (each with two pulses; MHNCI 180; Núcleo Picinguaba; M. R. Bornschein). (D) Example of one note group with four notes (each with two pulses; MHNCI 165). (E) Example of one note group with three notes (the first with three pulses and the remaining with two pulses; MHNCI 166; Estação Biológica de Boracéia, municipality of Salesópolis, São Paulo; M. R. Bornschein). (F) Example of one note group with three notes, with each note preceded by an attenuated note with one pulse (marked with white arrows; MHNCI 183). Spectrograms are produced with Hann window, overlap of 50%, and FFT size of 16,384 points in (A), 128 points in (B) and 256 points in (C)–(F).

These call descriptions do not allow for a reasonable comparison because they are not necessarily considering the same phenomenon. That is, when it was mentioned that *B. hermogenesi* call can be simple or composed (Verdade et al., 2008), it was being said, according to the note-centered approach (Köhler et al., 2017), that its call can have isolated notes or note groups, but the total number of notes in the entire *B. hermogenesi* call was not mentioned. In turn, when mentioning that the *B. sulfuratus* call has 4–7 notes (Condez et al., 2016), this represents the total number of notes in the call under note-centered approach (sensu Köhler et al., 2017) and that all are isolated notes (see Condez et al., 2016). This is one notorious distinctions between the calls of *B. sulfuratus* and *B. hermogenesi*: the former presents only isolated notes (Fig. 3) and the latter presents isolated notes and note groups (Fig. 4), with note groups having 2–7 notes, according Verdade et al. (2008), or 2–6 notes, according to our samples (Tables 2 and 3). Other particularities of the call of *B. hermogenesi* in relation to the one of *B. sulfuratus* is the high number of notes per call (≥24) and the presence of “attenuated notes” (Fig. 4F), while in the latter the call has few notes per call (≤8) without attenuated notes (Tables 2 and 3). We introduced attenuated notes as a new parameter, provisionally named, to describe weak notes issued before the notes along the calls of *B. hermogenesi*, more strongly perceived in spectrograms than in oscillograms (Fig. 4F). Due to this attenuated condition and difficulty in perceiving these notes, we did not include them as being part of note groups. We detect the presence of one attenuated note emitted before notes from both isolated notes and note groups, all of which from only three calls (MHNCI 167, MHNCI 183, MHNCI 215; Table 2).

**Table 2:** Structure of the advertisements calls recording between the geographical distribution of flea toads at some point identified as *B. sulfuratus*, *B. hermogenesi*, and as an unidentified related species.

Individuals (Ind) and call deposit number	Call structure	A	B
*B. sulfuratus*			
Ind 01 (MHNCI 123), ex 01	14, 11, 11, 11, 10, 9, 8	0	
Ind 01 (MHNCI 123), ex 02	12, 10, 11, 10, 10, 9, 8	0	
Ind 01 (MHNCI 123), ex 03	12, 11, 10, 9, 10, 9, 8	0	
Ind 01 (MHNCI 123), ex 04	14, 11, 10, 10, 10, 10, 8	0	
Ind 02 (MHNCI 124), ex 01	10, 7, 6	0	
Ind 02 (MHNCI 124), ex 02	6, 6, 6, 6	0	
Ind 02 (MHNCI 124), ex 03	9, 7, 7, 7	0	
Ind 02 (MHNCI 124), ex 04	10, 7, 8, 7, 3	0	
Ind 02 (MHNCI 124), ex 05	6, 6, 7, 9, 7, 4	0	
Ind 02 (MHNCI 124), ex 06	10, 9, 8, 8, 8, 7	0	
Ind 02 (MHNCI 124), ex 07	10, 9, 8, 9, 9, 8, 7	0	
Ind 02 (MHNCI 124), ex 08	10, 7, 10, 8, 9, 8	0	
Ind 02 (MHNCI 124), ex 09	9, 7, 8, 8, 8, 7	0	
Ind 02 (MHNCI 124), ex 10	10, 8, 7, 7, 8	0	
Ind 03 (MHNCI 125), ex 01	12, 10, 9, 9, 9, 8	0	
Ind 03 (MHNCI 125), ex 02	13, 9, 10, 10, 9, 8	0	
Ind 03 (MHNCI 125), ex 03	10, 9, 9, 9, 9, 9	0	
Ind 03 (MHNCI 125), ex 04	13, 9, 10, 9, 10, 8	0	
Ind 03 (MHNCI 125), ex 05	13, 10, 10, 10, 9, 9	0	
Ind 03 (MHNCI 125), ex 06	11, 9, 10, 10, 9, 8	0	
Ind 03 (MHNCI 125), ex 07	11, 9, 9, 9, 8	0	
Ind 03 (MHNCI 125), ex 08	12, 9, 9, 9, 9, 8	0	
Ind 04 (MHNCI 126), ex 01	?, ?, 9, 8, 8	0	
Ind 04 (MHNCI 126), ex 02	7, 8, 8, 8, 7	0	
Ind 04 (MHNCI 126), ex 03	6, 8, 7, 7, 7	0	
Ind 04 (MHNCI 126), ex 04	6, 8, 8, 8, 8	0	
Ind 04 (MHNCI 126), ex 05	6, 7, 7, 7, 7	0	
Ind 04 (MHNCI 126), ex 06	5, 7, 7, 8, 7, 6	0	
Ind 05 (MHNCI 127), ex 01	?, ?, ?, ?	0	
Ind 05 (MHNCI 127), ex 02	?, ?, ?, ?	0	
Ind 05 (MHNCI 127), ex 03	5, 6, 6, 6, 5	0	
Ind 05 (MHNCI 127), ex 04	?, ?, ?, ?, ?	0	
Ind 05 (MHNCI 127), ex 05	?, ?, ?, ?, ?, ?	0	
Ind 05 (MHNCI 127), ex 06	?, ?, ?, ?, ?	0	
Ind 05 (MHNCI 127), ex 07	7, 8, 8, 8, 7	0	
Ind 06 (MHNCI 128), ex 01	11, 10, 10, 9, 8	0	
Ind 06 (MHNCI 128), ex 02	11, 10, 10, 9, 8	0	
Ind 06 (MHNCI 128), ex 03	11, 10, 9, 10, 8	0	
Ind 06 (MHNCI 128), ex 04	12, 10, 9, 9, 8	0	
Ind 06 (MHNCI 128), ex 05	11, ?, ?, ?	0	
Ind 06 (MHNCI 128), ex 06	11, 10, 9, 8, 7	0	
Ind 06 (MHNCI 128), ex 07	11, 10, 9, 9, 9	0	
Ind 07 (MHNCI 129), ex 01	10, 8	0	
Ind 07 (MHNCI 129), ex 02	12, 8	0	
Ind 07 (MHNCI 129), ex 03	10, 8	0	
Ind 07 (MHNCI 129), ex 04	10, 8, 8	0	
Ind 07 (MHNCI 129), ex 05	10, 8, 7	0	
Ind 08 (MHNCI 129), ex 01	6, 5, 4, 4	0	
Ind 08 (MHNCI 129), ex 02	9, 9, 9, 9	0	
Ind 08 (MHNCI 129), ex 03	11, 8, 9, 9, 9, 9, 9	0	
Ind 08 (MHNCI 129), ex 04	9, 9, 7, 7, 9, 9	0	
Ind 09 (MHNCI 129)	10, 9, 9, 9, ?, 9, 8	0	
Ind 10 (MHNCI 130), ex 01	10, 7, 7, 6	0	
Ind 10 (MHNCI 130), ex 02	8, 9, 7	0	
Ind 11 (MHNCI 130), ex 01	?, ?, ?, ?, ?, ?	0	
Ind 11 (MHNCI 130), ex 02	?, ?, ?, ?, ?, ?, ?	0	
Ind 11 (MHNCI 130), ex 03	?, ?, ?, ?, ?, ?	0	
Ind 11 (MHNCI 130), ex 04	?, ?, ?, ?, ?	0	
Ind 11 (MHNCI 130), ex 05	11, 10, 9, 9, 9, 9, 8	0	
Ind 11 (MHNCI 130), ex 06	12, 9, 9, 9, 9, 9, 8	0	
Ind 11 (MHNCI 130), ex 07	11, 10, 9, 9, 9, 8	0	
Ind 11 (MHNCI 130), ex 08	11, 9, 8, 9, 8, 8,	0	
Ind 11 (MHNCI 130), ex 09	?, ?, 9, 9, ?, 8	0	
Ind 11 (MHNCI 130), ex 10	?, 9, 8, ?, 8, 8	0	
Ind 12 (MHNCI 131), ex 01	7, 6, 6, 5, 5, 4	0	
Ind 12 (MHNCI 131), ex 02	7, 6, 5, 6, 7, 5	0	
Ind 12 (MHNCI 131), ex 03	8, 6, 6, 6, 6, 5	0	
Ind 13 (MHNCI 132), ex 01	10, 7, 7, 7	0	
Ind 13 (MHNCI 132), ex 02	9, 8, 8, 8, 8	0	
Ind 13 (MHNCI 132), ex 03	10, 8, 8, 8, 8	0	
Ind 13 (MHNCI 132), ex 04	10, 9, 9, 9, 8	0	
Ind 13 (MHNCI 132), ex 05	10, 9, 9, 9, 9	0	
Ind 13 (MHNCI 132), ex 06	10, 9, 9, 9, 8	0	
Ind 13 (MHNCI 132), ex 07	10, 9, 9, 9, 9	0	
Ind 13 (MHNCI 132), ex 08	11, 9, 9, 9, 9	0	
Ind 13 (MHNCI 132), ex 09	10, 9, 8, 9, 9	0	
Ind 13 (MHNCI 132), ex 10	11, 9, 8, 9, 8	0	
Ind 13 (MHNCI 132), ex 11	10, 9, 10, 8	0	
Ind 13 (MHNCI 132), ex 12	10, 8, 8, 8	0	
Ind 14 (MHNCI 133), ex 01	?, ?, ?, ?	0	
Ind 14 (MHNCI 133), ex 02	?, ?, ?, ?	0	
Ind 14 (MHNCI 133), ex 03	?, ?, ?, ?, ?, ?	0	
Ind 14 (MHNCI 133), ex 04	?, ?, ?, ?, ?, ?	0	
Ind 14 (MHNCI 133), ex 05	?, ?, ?, ?, ?, ?	0	
Ind 14 (MHNCI 133), ex 06	11, 10, 9, 11, 9	0	
Ind 14 (MHNCI 133), ex 07	?, ?, 10, 9, 8	0	
Ind 14 (MHNCI 133), ex 08	8, 9, 9, 9, ?	0	
Ind 14 (MHNCI 133), ex 09	?, ?, ?, ?	0	
Ind 15 (MHNCI 134)	9, 7, 7, 7, 6, 6	0	
Ind 16 (MHNCI 135), ex 01	5, 5, 5, 5	0	
Ind 16 (MHNCI 135), ex 02	?, ?, ?, ?, ?	0	
Ind 17 (MHNCI 136), ex 01	11, 8, 7, 8, 7	0	
Ind 17 (MHNCI 136), ex 02	12, 9, 8, 8, 8	0	
Ind 17 (MHNCI 136), ex 03	12, 9, 8, 8, 8	0	
Ind 17 (MHNCI 136), ex 04	12, 9, 8, 7	0	
Ind 17 (MHNCI 136), ex 04	10, 9, 8, 5	0	
Ind 17 (MHNCI 136), ex 06	10, 8, 5, 3	0	
Ind 17 (MHNCI 136), ex 07	10, 8, 5	0	
Ind 17 (MHNCI 136), ex 08	9, 8, 6	0	
Ind 17 (MHNCI 136), ex 09	8, 8, 7	0	
Ind 17 (MHNCI 136), ex 10	9, 8, 7, 5	0	
Ind 18 (MHNCI 137), ex 01	6, 7, 6, 2	0	
Ind 18 (MHNCI 137), ex 02	6, 7, 6, 2	0	
Ind 18 (MHNCI 137), ex 03	?, 7, 7, 6	0	
Ind 18 (MHNCI 137), ex 04	8, 7, 8, 7	0	
Ind 19 (MHNCI 217), ex. 01	?, ?, 10, 10, 9	0	
Ind 19 (MHNCI 217), ex. 02	9, 10, 10, 9, 10	0	
Ind 20 (MHNCI 218), ex 01	?, 10, 10, ?, ?, ?	0	
Ind 20 (MHNCI 218), ex 02	?, ?, ?, ?, ?, ?	0	
Ind 21 (MHNCI 219), ex 01	9, 7, 7	0	
Ind 21 (MHNCI 219), ex 02	9, 7, 7, 6	0	
Ind 21 (MHNCI 219), ex 03	9, 7, 7, 7	0	
Ind 21 (MHNCI 219), ex 04	9, 9, 8, 8, 8	0	
Ind 21 (MHNCI 219), ex 05	10, 8, 8, 8, 8, 8, 8	0	
Ind 21 (MHNCI 219), ex 06	10, 9, 9, 8, 8, 8	0	
Ind 21 (MHNCI 219), ex 07	10, 9, 9, 8, 8, 8	0	
Ind 21 (MHNCI 219), ex 08	10, 9, 9, 9, 8	0	
Ind 21 (MHNCI 219), ex 09	10, 9, 9, 9, 9, 9, 9, 8	0	
Ind 21 (MHNCI 219), ex 10	9, 9, 8, 8	0	
Ind 21 (MHNCI 219), ex 11	10, 8, 7	0	
Ind 21 (MHNCI 219), ex 12	10, 8, 6	0	
Ind 21 (MHNCI 219), ex 13	9, 7, 6	0	
Ind 21 (MHNCI 219), ex 14	9, 8, 7	0	
Ind 21 (MHNCI 219), ex 15	10, 8, 7	0	
Ind 21 (MHNCI 219), ex 16	10, 8, 7	0	
Ind 21 (MHNCI 219), ex 17	10, 8, 7	0	
Ind 21 (MHNCI 219), ex 18	10, 8, 7	0	
Ind 21 (MHNCI 219), ex 19	10, 9, 8	0	
Ind 21 (MHNCI 219), ex 20	10, 9, 8	0	
Ind 21 (MHNCI 219), ex 21	10, 9, 8, 8	0	
Ind 21 (MHNCI 219), ex 22	10, 9, 9, 8	0	
Ind 21 (MHNCI 219), ex 23	10, 9, 8	0	
Ind 21 (MHNCI 219), ex 24	10, 9, 8	0	
Ind 21 (MHNCI 219), ex 25	10, 9, 8	0	
Ind 22 (MHNCI 220), ex 01	11, 8, 7, 7, 7, 7	0	
Ind 22 (MHNCI 220), ex 02	10, 8, 7, 7, 8, 8	0	
Ind 22 (MHNCI 220), ex 03	9, 8, 7, 7, 8, 7	0	
Ind 22 (MHNCI 220), ex 04	9, 8, 7, 8, 7, 7	0	
Ind 22 (MHNCI 220), ex 05	10, 8, 8, 8, 8, 8	0	
Ind 22 (MHNCI 220), ex 06	9, 8, 8, 8, 8, 8	0	
Ind 22 (MHNCI 220), ex 07	10, 8, 8, 8, 8, 8	0	
Ind 22 (MHNCI 220), ex 08	10, 8, 8, 8, 8, 8	0	
Ind 22 (MHNCI 220), ex 09	10, 8, 8, 8, 8	0	
Ind 22 (MHNCI 220), ex 10	10, 8, 9, 8, 8, 8	0	
Ind 22 (MHNCI 220), ex 11	10, 8, 7, 8, 8, 7	0	
Ind 22 (MHNCI 220), ex 12	10, 8, 8, 8, 8, 8	0	
Ind 22 (MHNCI 220), ex 13	10, 8, 8, 8, 7, 7	0	
Ind 22 (MHNCI 220), ex 14	10, 8, 8, 8, 8, 6	0	
Ind 22 (MHNCI 220), ex 15	10, 8, 8, 8, 8, 7	0	
Ind 22 (MHNCI 220), ex 16	9, 8, 7, 8, 7	0	
Ind 22 (MHNCI 220), ex 17	10, 9, 7, 8, 7	0	
Ind 23 (MHNCI 221), ex 01	8, 7, 6, 7, 6, 5	0	
Ind 23 (MHNCI 221), ex 02	8, 7, 7, 7, 7, 4	0	
Ind 23 (MHNCI 221), ex 03	8, 7, 7, 7, 6	0	
Ind 23 (MHNCI 221), ex 04	8, 6, 7, 7, 6	0	
Ind 23 (MHNCI 221), ex 05	8, 7, 7, 7, 6	0	
Ind 23 (MHNCI 221), ex 06	8, 7, 8, 7, 7	0	
Ind 23 (MHNCI 221), ex 07	8, 8, 7, 7, 7	0	
Ind 23 (MHNCI 221), ex 08	8, 8, 8, 7, 7	0	
Ind 23 (MHNCI 221), ex 09	9, 8, 7, 8, 7	0	
Ind 23 (MHNCI 221), ex 10	9, 8, 8, 8, 7	0	
Ind 23 (MHNCI 221), ex 11	9, 8, 7, 7	0	
Ind 23 (MHNCI 221), ex 12	8, 7, 6	0	
Ind 23 (MHNCI 221), ex 13	9, 7, 7, 6	0	
Ind 23 (MHNCI 221), ex 14	8, 7, 7, 7, 7	0	
Ind 23 (MHNCI 221), ex 15	9, 7, 7, 7, 7	0	
Ind 23 (MHNCI 221), ex 16	8, 7, 8, 7, 6	0	
Ind 23 (MHNCI 221), ex 17	8, 8, 8, 7, 7	0	
Ind 23 (MHNCI 221), ex 18	9, 8, 7, 8	0	
Ind 23 (MHNCI 221), ex 19	9, 8, 8, 7, 7	0	
Ind 23 (MHNCI 221), ex 20	9, 8, 7, 7, 7	0	
Ind 23 (MHNCI 221), ex 21	8, 7, 7, 7, 7	0	
Ind 23 (MHNCI 221), ex 22	9, 8, 7, 7	0	
Ind 23 (MHNCI 221), ex 23	9, 7, 7, 6	0	
Ind 23 (MHNCI 221), ex 24	9, 7, 7, 7	0	
*B. hermogenesi* (Corcovado)			
Ind 01 (MHNCI 165)	2, 2, 2, 2, 2, 2, 2, 2, 2, 2, 2, 2, 2, 2, 2, 2, 2, 2, 2, 2, 2, 2, 2, 2, 2, 2, 2, 2, 2, (2-2), (2-2), (2-2), (2-2), (2-2-2), (2-2-2), (2-2-2), (2-2-2-2), (2-2-2), (2-2-2-2), (2-2-2), (2-2-2-2), (2-2-2-2), (2-2-2-2), (2-2-2-2), (2-2-2), (2-2-2), (2-2-2-2), (2-2-2), (2-2-2-2), (2-2-2-2), (2-2-2-2), (2-2-2), (2-2-2-2), (2-2-2), (2-2-2-2), (2-2-2-2), (2-2-2), (2-2-2-2), (2-2-2), (2-2-2), (2-2-2)	2	
*B. hermogenesi* (Estação Biológica de Boracéia)			
Ind 01 (MHNCI 166)	2, 2, 2, 2, 2, 2, 2, 2, 2, 2, 2, 2, 2, 2, 2, (2-2), 2, 2, 2, (2-2), (2-2), (2-2), (2-2), (2-2), (2-2), (2-2), (2-2), (2-2-2), (2-2-2), (2-2-2), (2-2-2), (2-2-2-1), (2-2-2-2), (2-2-2), (2-2-2), (2-2-2-2), (2-2-2-2), (2-2-2-2), (2-2-1-1), (2-2-2-2), (2-2-2-2), (2-2-2-2), (3-2-2-2), (2-2-2-2), (2-2-2), (2-2-2-2), (3-2-2-2), (2-2-2), (2-2-2), (2-2-2), (2-2-2), (3-2-2), (2-2-2)	6	
Ind 02 (MHNCI 167)	2, 1, 2, 2, 1, 2, 2, 2, 2, 1, 2, 2, 2, 2, 2, 2, 2, 2, 2, 2, 2, 2, 2, 2, (2-2), (2-2), (2-2), (2-2), (2-2), (2-2-2), (2-2-2), (2-2-2), (2-2-2), (2-2-1), (2-2-2), (2-2-1), (2-1-2-2), (2-2-1), (2-2-1-1), (2-2-2), (2-2-2), (2-2-2), (2-2-1-1), (2-1-1-_1-_2), (2-1-_1-_1), (2-1-_1-_1-_1-_1), (2-2-1)	7	
Ind 03 (MHNCI 168)	2, 2, 2, 2, 2, 2, 2, 2, 2, 2, 2, 2, 2, 2, 2, 2, 2, 2, 2, 2, (2-2), (2-2), (2-2), (2-2-2), (2-2-2), (2-2-2), (2-2-2), (2-2-2-2), (2-2-2-2-2), (2-2-2-2), (2-2-2), (2-2-2-2), (2-2-2-2), (2-2-2), (2-2-2-2), (2-2-2-2)	3	
Ind 04 (MHNCI 169)	1, 1, 1, 1, 1, 1, 1, 1, 1, 1, 1, 2, 2, ?, ?, 2, 2, 2, 2, (2-2), (2-2-2), (2-2), (2-2), (2-2), (2-2), (2-2), (2-?-?), (2-2-2), (2-2), (2-2-2), (2-2-2), (2-?-2), (2-?-2), (2-2-2-1), (2-2-1), (2-2-2-2), (?-?-?-?), (2-?-?-?), (2-1-1), (2-2-2), (2-2-2), (?-2-?), (2-1-2), (2-1-2)	3	
*B. hermogenesi* (Morro do Cantagalo)			
Ind 01 (MHNCI 222)	?, ?, ?, ?, 2, 2, ?, 2, 2, 2	?	X
Ind 02 (MHNCI 223)	2, (2-2-2), (2-2-2), (2-2-2), (2-2-2), (2-2-2-2), (2-2-2-2), (2-2-2-2), (2-2-2-2)	?	X
*B. hermogenesi* (Núcleo Cunha)			
Ind 01 (MHNCI 170), ex 01	2, 2, 2, 2, 2, 2, 2, 2, 2, 2, 2, 2, (2-2), 2, 2, (2-2), (2-2), (2-2), (2-2)	0	
Ind 01 (MHNCI 171), ex 02	1, 1, 1, 1, 2, 2, 2, 2, 2, 2, 2, 2, 2, 2, ?, 2, 2, 2, 2, 2, 2, 2, 2, 2, (2-2), 2, (2-2), (2-?), 2, (2-2), (2-2), (2-2), (2-2)	0	
*B. hermogenesi* (Núcleo Picinguaba)			
Ind 01 (MHNCI 172), ex 01	2, 2, 2, 2, 2, 2, 2, 2, 2, 2, 2, 2, 2, 2, 2, 2, 2, (2-2), (2-2), (2-2), (2-2), (2-2), (2-2), (2-2), (2-2), (2-2), (2-2-2), (2-2-2), (2-2-2), (2-2-2), (2-2-2), (2-2-2), (2-2-2), (2-2-2), (2-2-1), (2-2), (2-2), (2-2), (2-2)	?	
Ind 01(MHNCI 175), ex 02	1, 2, 1, 1, 2, 2, 2, 2, 2, 2, 2, 2, 2, 2, 2, 2, 2, 2, 2, 2, 2, (2-1), (2-2), (2-2), (2-2), (2-2), (2-2), (2-2), (2-2)	0	
Ind 02 (MHNCI 173), ex 01	2, 2, 2, 2, 2, 2, 2, (2-1), (2-2), (2-2), (2-2), (2-2), (2-2), (2-2), (2-2-1), (2-1-1), (2-2-2), (2-1-2), (2-2), (2-2-1), (2-2-1), (2-1), (2-1), (1-1), (1-1), (1-1), 1	4	
Ind 02 (MHNCI 177), ex 02	1, 2, 2, 2, 2, 2, 2, 2, 2, 2, 2, 2, 2, 2, (2-2), 2, (2-2), (2-2), (2-1), (2-2), (2-2), (2-2-2), (2-2-2), (2-2-2), (2-2-2), (2-2-1), (2-1), (2-2), (1-1), (1-1)	0	
Ind 02 (MHNCI 182), ex 03	(2-2), (2-2), (2-2), (2-2), (2-2), (2-2)	?	
Ind 03 (MHNCI 174), ex 01	2, 2, 2, 2, 2, 2, 2, 2, 2, 2, 2, 2, 2, 2, 2, 2, (2-2), (2-2), (2-2), (2-2), (2-2), (2-2), (2-2), (2-2), (2-2-2), (2-2-2), (2-2-2), (2-2), (2-2-2), (2-2-2), (2-2-2), (2-2-2), (2-2-2), (2-2), (2-2-2), (2-2-1), (2-2)	6	
Ind 03 (MHNCI 178), ex 02	2, 2, 2, 2, 2, 2, 2, 2, 2, 2, 2, 2, 2, (2-2), (2-2), (2-2), (2-2), (2-2), (2-2), (2-2), (2-2), (2-2), (2-2-2), (2-2-2), (2-2-2), (2-2-2), (2-2-2), (2-2-2), (2-2), (2-2-2)	5	
Ind 03 (MHNCI 180), ex 03	2, 2, 2, 2, 2, 2, 2, 2, (2-2), (2-2), (2-2), (2-2), (2-2), (2-2-2), (2-2), (2-2-2), (2-2-2), (2-2-2), (2-2-2), (2-2-2), (2-2-2), (2-2-2), (2-2-2), (2-2-2), (2-2-1), (2-2-2), (2-2-1), (2-2-1), (2-2-1), (2-2-1)	?	
Ind 03 (MHNCI 181), ex 04	?, 2, 2, 2, 2, 2, 2, 2, 2, 2, 2, 2, 2, 2, 2, 2, 2, 2, 2, (2-2), (2-2), (2-2), (2-2), (2-2), (2-2), (2-2), (2-2), (2-2), (2-2), (2-2-2), (2-2), (2-2), (2-2-2), (2-2-2), (2-2-2), (2-2-2), (2-2-2), (2-2-1)	1	
Ind 04 (MHNCI 176), ex 01	2, 2, 2, 2, 2, 2, 2, 2, 2, 2, 2, (2-2-1), (2-2-2), (2-2-1), (2-2-2), (2-2-1), (2-2-2), (2-2-1), (2-2-1), (2-2-1), (2-2-1), (2-2-1), (2-2), (2-2-1), (2-2-1), (2-2-1), (2-2), (2-2), (2-2)	3	
Ind 04 (MHNCI 179), ex 02	(2-2), (2-2-2), (2-2-1), (2-2-1), (2-2-1), (2-2-2), (2-2-1), (2-2), (2-1)	?	
Ind 04 (MHNCI 183), ex 03	1, 1, 1, 1, 1, 2, 2, 2, 2, 2, 2, 2, (2-1), (2-1), (2-1), (2-2), (2-2), (2-2), (2-2), (2-2), (2-2), (2-2-2), (2-2), (2-2), (2-2-2), (2-2-2), (2-2-1), (2-2-1), (2-_1-_2-_1-_2), (2-_1-_2-_1-_2), (2-_1-_2-_1-_2), (_1-_2-_1-_2-_1-_2), (_1-_2-_1-_2-_1-_2), (_1-_2-_1-_2-_1-_1), (2-_1-_2-1), (2-1)	0	
Ind 05 (MHNCI 184)	(2-2-2), (2-2-2), (2-2-2), (2-2-2), (2-2-2), (2-2-2), (2-2-1), (2-2-2), (2-2-1)	?	
Ind 06 (MHNCI 185)	1, 1, 2, 2, 1, 1, 2, 2, 2, 2, 2, (2-1), (2-1), 2, (2-1), 2, (2-2), (2-2), (2-2), (2-2), (2-2), (2-1), (2-1), (2-1), (2-2), (2-2), (2-2)	2	
Ind 07 (MHNCI 186)	1, 2, 2, 2, 2, 2, 2, 2, 2, 2, 1, 2, 2, 2, 2, 1, 2, 2, 2, 2, 2, (2-2), (2-2), (2-2), (2-2), (2-2), (2-2), (2-2), (2-2), (2-2), (2-2), (2-2), (2-2), (2-2), 2, 2	1	
Ind 08 (MHNCI 187)	(2-2-1), (2-2), (2-2), (2-2), (?-?-?), (2-2), (2-2), (2-2), (2-1), (2-2)	?	
*B. hermogenesi* (Parque Natural Municipal Nascentes de Paranapiacaba)			
Ind 01 (MHNCI 213)	(?-?-?), (?-?-?), (2-2-2), (2-2-2), (2-2-2), (2-2-2), (2-2-2), (2-2-2), (?-?-?), (?-?-?-?), (?-?-?-?), (?-?-?-?), (?-?-?-?), (1-1-1-1)	?	
Ind 02 (MHNCI 214)	1, 1, 1, 1, 1, 1, 1, 1, 1, (1-1), 1, 1, 1, 1, 1, 1, 1, 1, 1, 1, 1, 1, 1, 1, 1, 1, 1, 1 ,1, 2, 1, 1, 1, 2, 2, 2, 1, 2, 2, 2, 2, 1, 2, 2, 2, 1, 1, 1, 1, 1, 1, 1, 2, 1, 1, 2, 1, 2, 2, 2, 2, 2, 2, 2, 2, 2, 2, 2, 2, 2, 2, 2 ,2, 2, 2, 2, 2, 2, 2, 2, 2, 2, 2, 2, (2-2), 2, 2, 2, 2, (2-2), 2, 2	0	
Ind 03 (MHNCI 215)	1, 1, 1, 1, 1, 1, 1, 1, 1, 1, 1, 1, 2, 2, 1, 3, 2, 2, 2, 3, 2, 2, 2, 2, 1, 2, 1, 1, 2, 2, 2, 2, 2, 2, _1-_2, (2-2), (2-2), (2-2), (2-2), (2-2), (1-2-2), (2-2-2), (2-2-2), (1-2-2), (2-2-2), (2-2-2), (1-1-2), (2-2-2), (2-2-2), (2-2-2), (2-2-1-1), (2-1-1-1), (2-1-1-2), (2-2-1-1), (2-2-1-2), (2-2-1), (1-2-1-2), (2-2-2-1)	0	
Ind 04 (MHNCI 216)	1, 1, 1, 1, 1, 1, 1, 1, 1, 1, 1, 1, 1, 1, 1, 1, 1, 1, 1, 1, 1, 1, 1, 1, 2, 1, 1, 1, 1, 1, 1, 1, 2, 1, 2, 1, 1, 2, 2, 2, 1, 2, 2, 1, 2, 2, 2, 2, 2, (2-2), (2-2), 2, (2-2), (2-2), (2-2), (2-2), (2-2), (2-2), (2-2), (2-2-2), (2-2), (2-1-1), (1-2-1), (2-2-1), (2-1-1), (2-1-1), (2-2-1), (1-1-1-1), (2-1-1-1), (1-1)	0	
*B. hermogenesi* (Trilha do Ipiranga 50 m from the Rio Ipiranga)			
Ind 01 (MHNCI 188)	(2-2), (2-2), (2-2), (2-1-1), (2-1-1), (1-1-1-1), (1-1-1), (1-1-1-1), (1-1-1-1), (1-1-1), (1-1-1), (1-1-1), (1-1-1)	?	
Ind 02 (MHNCI 189)	2, 2, 2, 2, 2, 2, 2, 2, 2, 2, 2, 2, 2, 2, 2, 2, 2, 2, 2, ?, ?, 2, 2, (1-2), (2-2), (2-2), (2-2), (3-2), (2-2-2), (2-2), (2-2-2), (2-2-2), (2-2-2), (2-2-2), (2-2-2), (2-2-1), (2-2-2), (2-2), (2-2-2)	3	
Ind 03 (MHNCI 190)	2, 2, 2, 2, 2, 2, 2, 2, 2, 2, 2, 2, 2, 2, (2-2), (2-2), (2-2), (2-2), (2-2), (2-2), (2-2), (2-2), (2-2), (2-2-2)	3	
Ind 04 (MHNCI 191)	2, 2, 2, 2, 2, 2, 2, 2, 2, 2, 2, 2, 2, 2, 2, 2, 2, (2-2), (2-2), (2-2), (2-2), (2-2), (2-2), (2-2), (2-2), (2-2), (2-2), (2-2)	4	
Ind 05 (MHNCI 192)	2, 2, 2, 2, 2, 2, 2, 2, 2, 2, 2, 2, 2, 2, 2, 2, 2, 2, 2, 2, 2, 2, 2, 2, 2, 2, 2, 2, 2, 2, 2, 2, 2, 2, 2, 2, 2, 2, 2, 2, 2, 2, 2, 2, 2, 2, 2, 2, 2, 2, 2, 2, 2, 2, 2, 2, (2-2), (2-2), (2-2), (2-2), (2-2), (2-2), (2-2), (2-2), (2-2-2), (2-2), (2-2-2), (2-2-2), (2-2-2), (2-2-2), (2-2-2-2), (2-2-2-2), (2-2-2-1), (2-2-1-2), (2-2-2-1), (2-1-1-1-1), (2-1-1-1-1), (2-1-1-1-1), (2-2-2-1-1), (1-1-1-1-1), (1-1-1-1-1-1), (2-1-1-1-1), (2-2-1-1-1), (1-2-1-1-1), (1-1-1-1-1), (1-1-1-1-1), (1-1-1-1-1), (1-1-1-1-1), (1-1-1-1-1), (1-2-1-1-1), (1-1-1-1-1), (1-2-1-1-1), (1-1-1-1-1), (1-1-1-1-1), (1-1-1-1), (1-1-1-1), (1-1-1-1-1), (2-1-1-1-1), (2-1-2-1-1), (1-2-1-2-1), (2-1-1-2-1), (1-1-1-1-1), (1-2-1-1-2), (1-1-1-1-1), (1-1-1-1), (1-1-1-1-1)	3	
*Brachycephalus* sp. (Corcovado)			
Ind 01 (MHNCI 193)	(6-4), (6-4), (6-4), (6-4), (6-4), (6-4), (6-1)	?	
Ind 02 (MHNCI 194)	1, 2, 2, ?, 2	0	X
Ind 03 (MHNCI 195)	3, 3, 4, 3, 4, 4, 3, 3, 4, 4, 3, 4, 4, 4, 4, 4, 4, 4, 4, 4, 4, 4, 4, 5, 5, 4, 5, 4, 4, 4, (5-2), (5-4), (5-2), (5-3), (5-2), 6, 5, 4, (5-1), 5, (5-3), (5-3), (5-1), (6-4), (5-3), (5-4), (5-3), (5-3), (5-3), (5-3), (5-3), (5-4), (5-3), 6	?	
Ind 04 (MHNCI 196), ex 01	3, 3, 2, 3, ?, 3, ?, ?, ?, ?, ?, 4, 4, 4, 4, 3, 3, ?, 4, ?, 3, 4, 4, 5, 4, 4, 5, 4, 4, ?, ?, 5, 5, 5, 5, (4-3), (?-?), (6-4), (7-4), (9-4), (8-4), (9-5), (9-4), (10-5), (9-6), (11-5), (11-5), (8-5), (6-5), (6-4), (7-4), (6-4), 6, 5, 5, 5, 5, 5, 5, 5	3	
Ind 04 (MHNCI 200), ex 02	4, 4, 4, 5, 5, 4, 5, 4, 4, 4, 4, 5, 5, 5, (4-3), 5, 5, 6, (6-3), (7-4), (6-3), (7-3), (8-4), (7-4), (8-4), (?-?), (8-4), (9-4), (9-4), (8-5), (8-5), (8-4), (9-5), (7-4), (8-5), (8-4), (7-6), (6-5), (7-4), (6-5), (6-4), 6, 5, 7, 5, 4, 5	7	
Ind 05 (MHNCI 197)	(5-3), (5-4), 4, (3-3), (4-3), (4-3), 4, 4, 4, (3-3), 4, 3	?	
Ind 06 (MHNCI 198), ex 01	6, 10, 4, 10, 10, 12, (13-2), (8-3), (12-2), (9-3), 5, 7, 11, (9-4), (13-3), (14-5), (16-4), (15-5), (11-5), (9-4), (9-8), 4	4	
Ind 06 (MHNCI 199), ex 02^1^	5, 5, 4, 5, 5, 4, 3, 4, 5, 4	?	
Ind 07 (MHNCI 201)	4, 3, 4, 4, 5, 4, 4, 4, 4, 4, 5, 5, 5, 5, 5, 5, 5, 5, 6, (6-4), (7-4), (7-3), (7-4), (7-4), (7-4), (7-4), (7-5), (7-5), (7-5), (7-4), (7-2), (7-5), (7-5), (6-5), (6-5), (7-5), (7-5), 5, 6, 5, 6, 5, 6, 5, 6	4	
Ind 08 (MHNCI 202)	2, 3, 2, 3, 3, 3, 3, 3, 3, 3, 3	6	X
Ind 09 (MHNCI 203)	4, 4, 4, 4, 4, 4, 4, 4, 4, 4, 4, 4, (4-2), (4-2), (5-3), (4-3), (5-3), (5-3), 5, (5-3), (4-2), (5-3), (5-3), (4-3), (5-3), (5-3), 5, 4, 5, 5, 5, 5, 4, 4, 4, 4, 5, 4, 4, 4, 4, 5, 4, 4, 4, 4, 4, 4, 4, 3, 3	2	
Ind 10 (MHNCI 204)	?, ?, ?, ?, ?, ?, ?, 2, 2, 3, 3, 2, 2, 3, 3, 3, 3, ?, 3, 4, 4, 3, 4, 4, 4, 4, (4-2), (4-3), 4, (4-3), (4-2), 4, (4-3), (4-1), (4-2), (4-3), (4-2), 4, (4-3), (4-3), (4-3), 5, 3, 4, 4	2	
Ind 11 (MHNCI 205)	1, 1, 2, 2, 2, 1, 2, 2, 2, 2, 2, 1, 3, 2, 2, 2, 2, 2, 3, 2, 2, 3, 3, 3, 4, 3, 3, 3, 4, 3, 4, 4, 4, 4, 4, 4, 4, 4, 4, 4, 4, 4, 4, 4, 4, 4, 4, 4, 4, 4, 4, 4, 4, 4, 4, 4, 4, 4, 4, 4, 4, 4, 4, 4, 4, 4, 4, 4, 4, 4, 4, 4, 4, 4, 4, 3, 3, 4	2	
Ind 12 (MHNCI 205)	2, 2, 2, 2, 2, 2, 2, 1, 2, 2, 2, 2, 2, 2, 2, 2, 2, 2, 2, 2, 2, 3, 2, 2, 3, 3, 3, 3, 3, 3, 3, 2, 3, 3, 3, 3, 3, 3, 3, 3, 3, 3, 4, 2, 1	0	
*Brachycephalus* sp. (Trilha do Corisco)			
Ind 01 (MHNCI 206)	(?-?),(?-?),(?-?),(?-?),(5-?),(?-?),(?-?),(4-?),(?-?),(?-?),(?-?)	?	
Ind 02 (MHNCI 207)	3, (4-3), (4-3), (4-3), (4-3), (4-3), (4-3), (4-3), (4-3), (3-3), (3-3), (3-3), (4-3), (4-3), (3-3)	?	
Ind 03 (MHNCI 208)	4, 3, 4, 4, 4, 4, 4, 4, 4, 4, 4, 4, 4, 4, 4, 4, 5, 5, (4-4), 5, (4-3), 4, (4-4), (5-4), (4-4), (4-3), (4-4), 4, 5, 5, 5, (5-3), 4	?	
Ind 04 (MHNCI 209)	2, 3, 3, ?, 3, 3, 4, (3-3), (4-3), (4-3), (4-3), (3-3), (4-3), (4-3), (4-3), (3-3), (4-3), (4-3), (4-3), (3-3), (3-2), 3, (3-2)	?	
Ind 05 (MHNCI 210)	4, 4, 4, 4, 4, 4, 4, 4, (4-3), 4, (4-3), (4-3), 4, (4-3), (4-3), (4-3)	5	X
Ind 06 (MHNCI 211)	4, (4-3), (3-3), (4-3), (4-4), (4-3), (4-4), (3-3), (4-4), (4-4), (4-4), (4-4), (4-3), (4-3), (4-4), (4-4), (3-4-4), (3-4-3), (4-4), (4-4), (3-3-3), (4-3-3), (3-3-3), (3-2-3), (3-2-3), (3-3), (3-2)	?	
Ind 07 (MHNCI 212)	3, (3-2), (3-3), (3-3), (3-3), (3-3), (3-3), (3-3), (3-3), (3-3), (3-3)	?	

**Notes:**

1 Only the final part of the advertisement call was recorded.

Structure of the advertisements calls (AC) recording by the author between the geographical distribution of flea toads at some point identified as *Brachycephalus sulfuratus*, *B. hermogenesi*, and as an unidentified related species, southeastern and southern Brazil. Each number represents a note, while the numerical value indicates the number of pulses for each note. Numbers in normal font outside parentheses represent isolated notes and those in normal font between parentheses represents note groups. Numbers in subscript represents attenuated notes (see text for reasons why we do not consider it as forming note groups). Question marks (“?”) represents a note issued whose number of pulses could not be counted. Abbreviations: A = number of isolated notes we hear being emitted before recording the AC; B = AC emission probably interrupted due to the researcher movement in the field.

**Table 3:** Parameters distinguishing the advertisement calls of flea toads at some point identified as *B. sulfuratus* and *B. hermogenesi*, including call comparisons of a third flea toad (*Brachycephalus* sp.).

Parameter	*B. sulfuratus*	*B. hermogenesi*	*Brachycephalus*. sp. from Corcovado and Trilha do Corisco
Note-centered approach			
Number of notes per call	≤8	≥24	≥38
Calls composed only by isolated notes	x		
Calls present note groups		x	x
Presence of warming notes		x	x
Presence of attenuated notes		x	
Maximum number of pulses in isolated notes	14	2	12
Maximum number of pulses per note in note groups	—	3	16
Maximum number of notes in note groups	—	6^1^	3
Call-centered approach			
Number of notes per call	1	1	1
Calls composed only by isolated notes	—	—	—
Calls present note groups	—	—	—
Presence of warming notes	—	—	—
Presence of attenuated notes	—	—	—
Maximum number of pulses in isolated notes	—	—	—
Maximum number of pulses per note in note groups	—	—	—
Maximum number of pulses per note	14	3	16
Maximum number of notes in note groups	—	—	—

**Notes:**

1 Up to seven, according Verdade et al. (2008).

Parameters distinguishing the advertisement calls of flea toads at some point identified as *Brachycephalus sulfuratus* and *B. hermogenesi*, including call comparisons of a third flea toad (*Brachycephalus* sp.), originally identified as *B. hermogenesi*.

**Figure 5:**
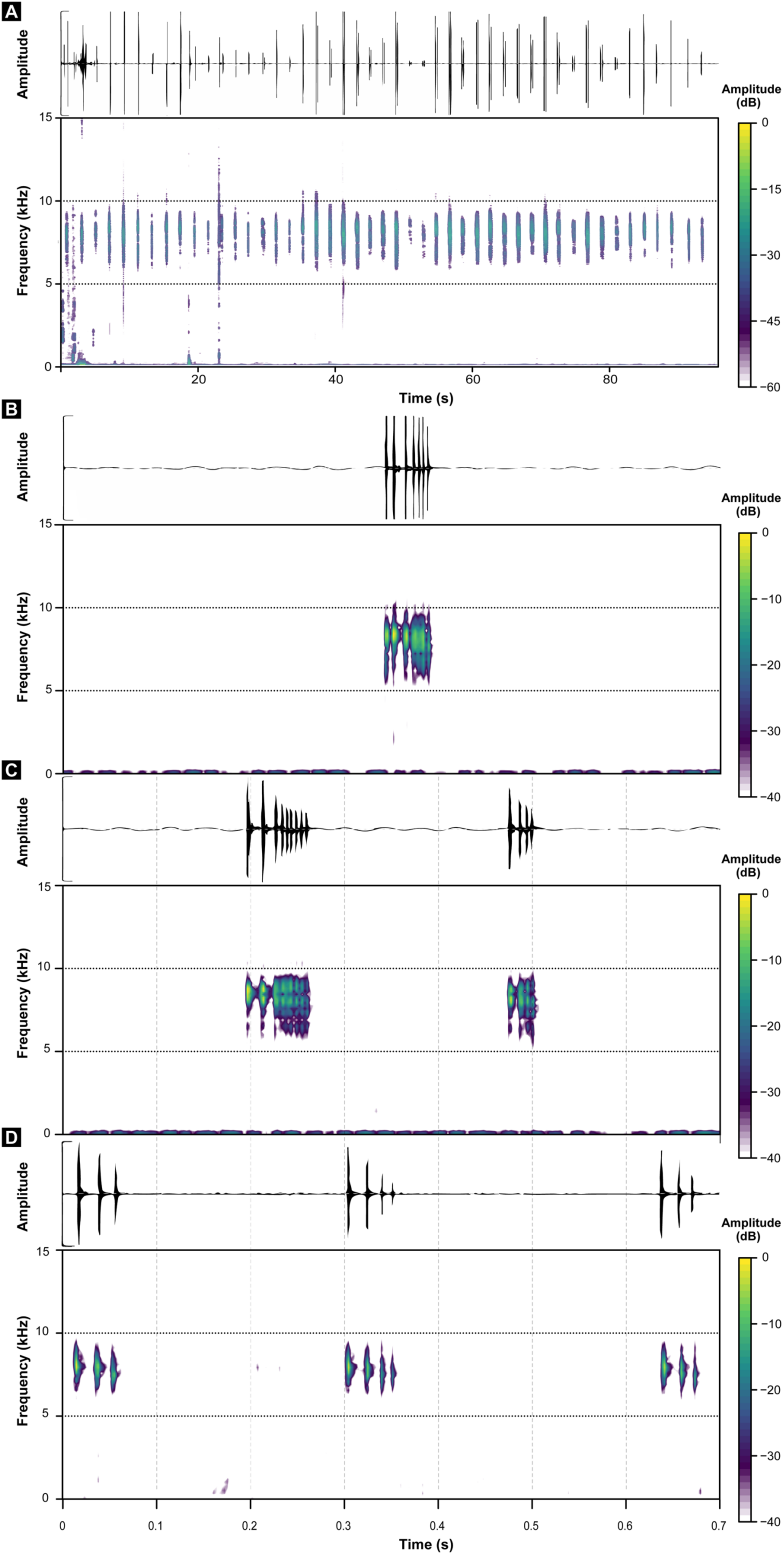
Oscillograms and spectrograms of *Brachycephalus* sp. (other than *B. sulfuratu*s and *B. hermogenesi*). (A) Example of one entire call with 71 notes recorded (MHNCI 200; Corcovado, municipality of Ubatuba, São Paulo; M. R. Bornschein). (B) Example of one isolated note with seven pulses (MHNCI 198; Corcovado; M. R. Bornschein). (C) Example of one note group with two notes (with nine and four pulses, respectively; MHNCI 198). (D) Example of one note group with three notes (the first note with three pulses and the remaining notes with four pulses; MHNCI 211; Trilha do Corisco, municipality of Paraty, Rio de Janeiro; L. F. Ribeiro). Spectrograms are produced with Hann window, overlap of 50%, and FFT size of 16,384 points in (A) and 256 points in (B)–(D).

Regarding number of pulses per note, *B. sulfuratus* was described as having 7–11 (Condez et al., 2016), but we found 2–14 (Table 1). Verdade et al. (2008) have not described the number of pulses of notes of *B. hermogenesi*, as stated by Condez et al. (2016: 50; “with 1–3 pulses”). However, as we demonstrated, the number of pulses per note for *B. hermogenesi* is indeed 1–3 (Table 2). We noticed that the calls of individuals of two localities previously attributable of *B. hermogenesi* differs from the descriptions above, by having notes with up to 16 pulses and two or rarely three notes in note groups (Fig. 5; Tables 1–3). These calls were from Trilha do Corisco, municipality of Paraty, Rio de Janeiro state, and Corcovado, municipality of Ubatuba, São Paulo state (see below; Table 1).

We erect as a diagnosis between *B. sulfuratus* and *B. hermogenesi* the few number of notes per call (≤8) with only isolated notes of *B. sulfuratus*, while in *B. hermogenesi* the advertisement call has a high number of notes (≥24) with the presence of isolated notes and note groups (see Table 3). In depth analysis of spectral and temporal parameters of the calls of *B. hermogenesi* will possibly bring other diagnostic parameters, as possibly the note rate, focus of a specific study in the future.

### Geographical occurrence records of *Brachycephalus sulfuratus* and *B. hermogenesi*

Based on our review of the 14 occurrence records of “*Brachycephalus* sp. nov. 1” from Pie et al. (2013), we conclude that the vouchered records correspond to *B. sulfuratus* (Table 1). Specimens from Pie et al. (2013) have yellow spots on their ventral side and advertisement calls with few notes and only isolated notes (as above). We treated unvouchered records of Pie et al. (2013) as *Brachycephalus* sp. (being probably *B. sulfuratus*; Table 1), with the exception of Castelo dos Bugres, due to the fact that, years later, Condez et al. (2016) collected specimens there, confirming the species’ identity as *B. sulfuratus*. We also determined previously unidentified *Brachycephalus* records from “Apiaí”, “Caratuval”, “Corvo” and “Fazenda Thalia” (Firkowski et al., 2016) as *B. sulfuratus* (Table 1) based on vouchered identification (specimens had yellow spots on their ventral region—see Fig. 1). The records of “*Brachycephalus* sp. 1” from Bornschein et al. (2016a) correspond to *B. sulfuratus* (Table 1): all but one of them are the same records as those records presented in Pie et al. (2013) and Firkowski et al. (2016) and were re-identified above. The only exception is the record of “*Brachycephalus* sp. 1” from RPPNSM, municipality of Guaraqueçaba, Paraná, identified as *B. sulfuratus* (Table 1) based on their call structure, with few notes and only isolated notes (MHNCI 133; Table 2). On the basis of this record, we reverted in favor of *B. sulfuratus* all other records of *B. hermogenesi* at RPPNSM (Pereira et al., 2010; Santos-Pereira et al., 2011, 2016; Santos-Pereira, Pombal & Rocha, 2018; Leivas et al., 2018; Table 1).

**Figure 6:**
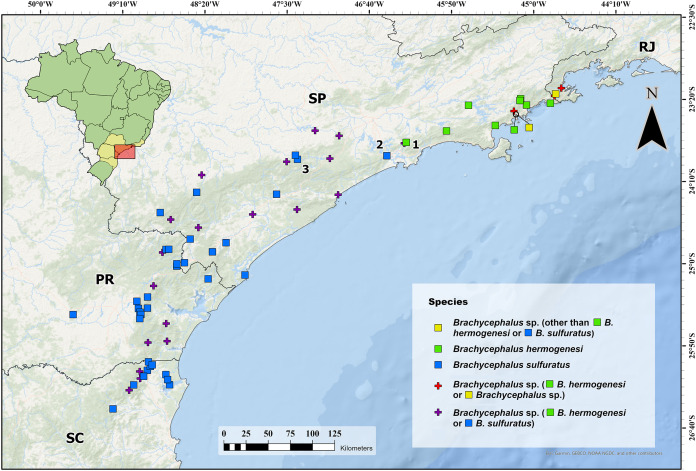
Current identification of records of flea toads that have been at some point identified as *Brachycephalus sulfuratus*, *B. hermogenesi*, and as an unidentified related species. Current identification of records of flea toads that have been at some point identified as *Brachycephalus sulfuratus*, *B. hermogenesi*, and as an unidentified related species, according to the compilation of localities and review of identifications shown in Table 1. We highlighted the southernmost record of *B. hermogenesi* confirmed (1—Parque Natural Municipal Nascentes de Paranapiacaba). We also highlight the northernmost confirmed records of *B. sulfuratus* (2—Núcleo Itutinga-Pilões and 3—near the Jurupará dam). Abbreviations: RJ = Rio de Janeiro; SP = São Paulo; PR = Paraná; SC = Santa Catarina. Map image is the intellectual property of Esri and is used herein under license. Copyright © 2020 Esri and its licensors. All rights reserved.

**Figure 7:**
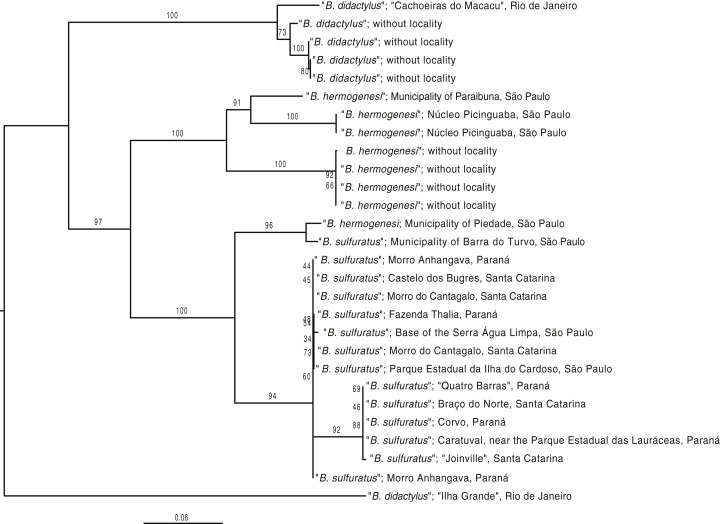
Phylogenetic tree based on a concatenated dataset of all mitochondrial 12S and 16S mitochondrial loci available on GenBank for specimens of the *B. didactylus* species group. Phylogenetic tree based on a concatenated dataset of all mitochondrial 12S and 16S mitochondrial loci available on GenBank for specimens of the *B. didactylus* species group (Table S1). The tree was rooted by its midpoint. Whenever possible, the corresponding localities available on their GenBank records were standardized based on the toponyms indicated in Table 1. Notice that the specimen originally identified as *B. hermogenesi* from the Municipality of Piedade (Condez, Sawaya & Dixo, 2009, Clemente-Carvalho et al., 2011), was reverted to *B. sulfuratus* (Table 1). Branch values correspond to bootstrap support.

Some previous studies reporting “*Brachycephalus hermogenesi*” (Giaretta & Sawaya, 1998; Dixo & Verdade, 2006; Verdade et al., 2008; Condez, Sawaya & Dixo, 2009; Verdade, Rodrigues & Pavan, 2009) from São Paulo do not provide enough morphological evidence or other details to allow us to reassess their original identification by us (Table 1; Fig. 6). Therefore, we propose that these identifications should be reverted as *Brachycephalus* sp. (being *B. hermogenesi* or *B. sulfuratus*). One of these records involves “*B. hermogenesi*” from the municipality of Piedade, state of São Paulo, of Condez, Sawaya & Dixo (2009) and Clemente-Carvalho et al. (2011), whose genetic sequence is deposited in GenBank (HQ435682.1 and HQ435709.1; Table 1). The corresponding voucher was obtained by T. H. Condez, 2016, personal communication in her study on the same location (Condez, Sawaya & Dixo, 2009). Phylogenetic analyses suggest that it might actually be *B. sulfuratus*, which was placed on the tree together with a specimen from the Municipality of Barra do Turvo, in an early-diverging branch of the *B. sulfuratus* clade on the tree (Fig. 7).

There are some specimens in the original description of *B. sulfuratus* (Condez et al., 2016), from six different localities, cited as “*B. hermogenesi*” in the appendix. It is possible that all of these records were identified based on preserved material, which does not allow for proper identification, as indicated above. Therefore, we also propose that those identifications should be considered as *Brachycephalus* sp. (being *B. hermogenesi* or *B. sulfuratus*; Table 1; see also Bornschein et al., 2016a).

There is a particular specimen, ZUEC 16602 (see “Introduction”), also examined by us, collected in the state of Paraná, that was first identified as “*Brachycephalus* aff. *hermogenesi*” (Cunha, Oliveira & Hartmann (2010), later as “*B. hermogenesi*” (Oliveira et al. (2011), “*Brachycephalus* sp. nov. 1” (Pie et al., 2013), “*Brachycephalus* sp. 1” (Bornschein et al., 2016a), and, finally as “*B. sulfuratus*” (Condez et al., 2016)). There is also the possibility that this specimen may not have been properly analyzed with respect to coloration in life, preventing the precise identification. Therefore, we also propose that this identification should be reverted to *Brachycephalus* sp. (being probably indeed *B. sulfuratus*; Table 1).

Advertisement calls analyzed of samples from Trilha do Corisco and Corcovado (in *part*.), two localities previously considered as occurrence of *B. hermogenesi* (Giaretta & Sawaya, 1998; Verdade et al., 2008; Pie et al., 2013, 2018b; Bornschein et al., 2016a; Bornschein, Pie & Teixeira, 2019; Table 1), have reveal substantial differences to made us to considerer that represents other species, unidentified, but not *B. sulfuratus* (Tables 2 and 3). The call from this third species has two notes forming note groups, exceptionally three, and includes notes with a high number of pulses (up to 16; Tables 2 and 3). Specimens we collected at Corcovado (MHNCI 10823–5) confirmed that they belong to the *B. didactylus* species group (sensu Pie et al., 2018b). Three adjacent locations based on unvouchered records, Morro Cuscuzeiro. Morro do Corcovado, and municipality of Paraty (Table 1), were referred to as *Brachycephalus* sp., perhaps *Brachycephalus* sp. from Trilha do Corisco and Corcovado (Table 1; Fig. 6). This third flea toad *Brachycephalus* sp. occurs in sympatry with *B. hermogenesi* in Corcovado, as proved by our recordings (*B. hermogenesi*: MHNCI 165; *Brachycehalus* sp.: MHNCI 165–205). The phylogenetic analysis revealed that the specimen from Municipality of Paraibuna is indeed *B. hermogenesi* (Table 1), being placed with other specimens of the species collected at the type locality (Fig. 7).

## Discussion

Based on our analyses, characters previously used as diagnostic for *B. sulfuratus* were quite variable and overlapped with those of *B. hermogenesi*. Moreover, the examination of specimens deposited in the collections MHNCI and ZUEC support this claim. Currently, differences in the call structure—number of notes per call and presence/absence of note groups - is proposed here as the only available sources of evidence supporting the distinction between *B. sulfuratus* and *B. hermogenesi*. Even in the field its advertisement calls are very distinct to the human ear and easily distinguishable. The advertisement calls of *B. sulfuratus* sounds like a “tríííííí, tríííííí, tríííííí, tríííííí, tríííí”, whereas the calls of *B. hermogenesi* from it type locality sound like a “tíc, tíc, tíc, tíc-tíc, tíc-tíc-tíc, tíc-tíc-tíc, tíc-tíc-tíc, …”. These transliterations represent isolated notes or note groups (each note separated by comma and note group by hyphen) with distinct durations (= transliteration size) related to the number of notes in the call. This diagnosis between *B. sulfuratus* and *B. hermogenesi* is only feasible under the note-centered approach. Considering their calls under the call-centered approach, there would be no diagnosis to be proposed between them at this moment, because each note would represent a call (Table 3). To the best of our knowledge, this is the first case in which the diagnosis between species of any *Brachycephalus* is made solely by characters of their advertisement call.

The first notes emitted from an advertising call by *B. hermogenesi* are usually hardly noticed in the recording and equally difficult to hear in the field. This is the reason why we rarely record the first emissions and many recordings recorded the advertisement call already in progress. These weak starting notes of an advertisement call were called warming notes (Bornschein et al., 2018; Table 3), assuming that they would reflect the individual’s preparation process to the level of excitement required for the issuance of “typical” strongest notes. Like warming notes, attenuated notes could prepare the individual to issue the immediately subsequent notes at a higher level of arousal.

The recognition of the existence of warming notes and attenuated notes, as well as the existence of note groups for understanding the richness of characters in *Brachycephalus* calls (see also above), consolidate the benefit of the note-centered approach over the call-centered approach in describing calls of species of this genus (Bornschein et al., 2018). The note-centered approach way for description the calls of *B. hermogenesi* also reinforces the hypothesis of complexity increment along note emissions (Bornschein et al., 2018), with the incorporation of note groups during the call emission. These structural particularities would not be perceived under the call-center approach. Under this approach, they would be perceived as a simple intraspecific variation in calls

The advertisement calls of *B. hermogenesi* show the same pattern as species from the *B. pernix* group (Bornschein et al., 2018, 2019; Pie et al., 2018b; Monteiro et al., 2018b, 2018a), which includes most species of southern Brazil, whereas the call of *B. sulfuratus* resembles the call of *B. vertebralis* (MRB, unpublish data), for example, from the *B. ephippium* group, which includes most species from the state of São Paulo to the north up to Espírito Santo and Minas Gerais.

We now confirm the absence of occurrence records of *B. hermogenesi* in southern Brazil and the presence of *B. sulfuratus* as far north as the east of São Paulo city, only 25 km in straight line from the southernmost site of a confirmed record of *B. hermogenesi* (Parque Natural Municipal Nascentes de Paranapiacaba; Fig.6; Table 1). Most unidentified records (Table 1) represent one or the other of these two species. In fact, it is likely that in southern Brazil only the flea toad *B. sulfuratus* occurs. In this region, our research group has been working with two anuran genera (*Brachycephalus* and *Melanophryniscus*) since 2009, focusing on their distribution, ecology and conservation (Pie et al., 2013; Bornschein et al., 2015, 2016a; Bornschein, Pie & Teixeira, 2019), and thus are particularly aware of *Brachycephalus* calls wherever we do field work and yet we never recorded *B. hermogenesi* calls in southern Brazil.

In addition, we also underscore the absence of records of *B. sulfuratus* in northern Santa Catarina in some well sampled localities. For example, we obtained no records for *B. sulfuratus* in Morro Boa Vista (26°30′58″S, 49°03′14″W), on the border between the municipalities of Jaraguá do Sul and Massaranduba, where we described *B. albolineatus* (Bornschein et al., 2016b), Morro do Baú (26°47′58″S, 48°55′47″W), municipality of Ilhota and Morro Braço da Onça (26°44′58″S, 48°55′41″W), municipality of Luiz Alves, where we report *B. fuscolineatus* (Ribeiro et al., 2015; Bornschein, Teixeira & Ribeiro, 2019), Morro do Cachorro (26°46′42″S, 49°01′57″W), on the border between the municipalities of Blumenau, Gaspar, and Luiz Alves, where we described *B. boticario* (Ribeiro et al., 2015), and Morro Santo Anjo (26°37′41″S, 48°55′50″W), municipality of Massaranduba, where we described *B. mirissimus* (Pie et al., 2018b). It is possible that the southern limit of the geographical distribution of *B. sulfuratus* occurs at the Morro do Garrafão (Table 1).

Contrary to what is found in southern Brazil, the distribution of flea toads in the states of São Paul and southern Rio de Janeiro are poorly known. Our findings indicate the presence of a third flea toad species at the border between São Paulo and Rio de Janeiro states, at least occurring in Corcovado, São Paulo, and Trilha do Corisco, municipality of Paraty, Rio de Janeiro. Corcovado, however, is one locality of paratypes of *B. hermogenesi* and Paraty were also cited as a place of occurrence of *B. hermogenesi* in the original description of this species (Giaretta & Sawaya, 1998). The species of the *B. didactylus* group that occurs closest to Rio de Janeiro/São Paulo border, excluding *B. hermogenesi* and *B. sulfuratus*, is *B. didactylus*, in Vila Dois Rios, Ilha Grande, municipality of Angra dos Reis, Rio de Janeiro (Bornschein, Pie & Teixeira, 2019). The Trilha do Corisco is distant from Vila Dois Rios 59 km in a straight line.

As we demonstrate in our analyses, there is no confirmed overlap in the distribution of *B. hermogenesi* and *B. sulfuratus*, and their geographical replacement occurs in southeastern of São Paulo city, without apparent barriers. There are other examples of discontinuity of the geographical distribution between congeneric species throughout the Atlantic Forest from southeastern to southern Brazil in southeastern São Paulo city, as in the montane bird *Scytalopus speluncae* (taxonomy sensu Maurício et al. (2010)). Maurício (2005) stated that populations of *S. speluncae* from the southeastern of the city of São Paulo to the south of the species distribution represent a distinct species yet to be named, and he treated it as “Southern *Scytalopus speluncae*” (this scenario of southern population of this bird as a new species was supported by other studies (Bornschein et al., 2007; Mata et al., 2009; Maurício et al., 2014; Pulido-Santacruz et al., 2016)). In the region around the southeastern of São Paulo city, cases of hybridization of subspecies or lineages have been reported for at least four species of birds (Pinto, 1941; Silva & Stotz, 1992; Cabanne, Santos & Miyaki, 2007; D’horta et al., 2011; see also Dantas et al., 2015). In the state of São Paulo there is another discontinuity which is associated with intraspecific differentiation or even sister species of frogs (Fitzpatrick et al., 2009; Thomé et al., 2010; Amaro et al., 2012) and snakes (Grazziotin et al., 2006).

The correspondence between the distribution of the congeneric species in question with the limits of the Serra do Mar is intriguing, given that during the last 20 million years there was no obvious uplift in the region (Gontijo-Pascutti et al., 2012). This time scale is considerably older than the inferred cladogenesis events and therefore geological processes could not have been the primary cause of their divergence, given that *Brachycephalus* toads and *Scytalopus* birds of São Paulo, Paraná, and Santa Catarina originated less than 2–5 million years ago (Pie et al., 2018a and Pulido-Santacruz et al., 2016, respectively). Likewise, recent neotectonic activities (Late Pleistocene-Holocene) are restricted to the faults and stress regimes (Hasui, 1990; Saadi, 1993; Ricommini & Assumpção, 1999) and, therefore, also could not have generated the diversification pattern of widely distributed terrestrial species. It is important to note that Thomé et al. (2010), studying the toad *Rhinella crucifer* from the eastern portion of Brazil, associate one genetic break found in eastern São Paulo to neotectonic barriers, specifically the Cubatão shear zone and the Guapiara lineament. However, these are ancient geotectonic activities, from Proterozoic to Cambrian (with Phanerozoic reactivation) and Mesozoic, respectively (Ferreira et al., 1981; Sadowski, 1991; Almeida & Carneiro, 1998; see also Ricommini & Assumpção, 1999). In addition, studies have proposed speciation by vicariance caused by relatively recent events, such as river barriers (Amaral et al., 2013), sea level variation (Grazziotin et al., 2006; Fitzpatrick et al., 2009), and forest refugia (Fitzpatrick et al., 2009; Thomé et al., 2010; D’horta et al., 2011; Amaral et al., 2013). The largest river around the disruption of the geographical distribution of *B. sulfuratus* and *B. hermogenesi* is the Rio Ribeira do Iguape, which intersects the Serra do Mar between São Paulo and Paraná States by continued erosive retreat (Almeida & Carneiro, 1998). Alternatively, the disruption of the Serra do Mar in that region originated from a tectonic depression associated with the asymmetric graben of the Sete Barras or Ribeira de Iguape (Melo et al., 1989; Gontijo-Pascutti et al., 2012). However, the formation of the present configuration of the Serra do Mar did not lead to isolation, given that *B. sulfuratus* occurs on both banks of the Ribeira do Iguape river. It is plausible that the origin of *B. sulfuratus* and *B. hermogenesi*, as well as the other examples mentioned above, might have resulted from climatic variations that promoted vicariance by forest cover disruption followed by the recovery of forest cover, presumably leading to secondary contact.

The region in the state of São Paulo, around the southeastern São Paulo city, should be further investigated. Records of flea toads in this region could be obtained as background sound in recordings of birds (e.g., recordings deposited in databases such as www.xeno-canto.org and www.wikiaves.com.br; Table 1). Verdade et al. (2008) made a similar suggestion: to search for records of *B. hermogenesi* in the background of recordings of birds from the Estação Biológica de Boracéia, in the case one wants to seek previous records of this flea toad in this highly sampled locality. As examples, calls of *B. sulfuratus* in Parque Estadual Intervales, municipality of Iporanga, state of São Paulo (Table 1), can be heard in recordings of the birds *Merulaxis ater* (XC80463 and XC18179) and *Eleoscytalopus indigoticus* (XC75544; available at www.xeno-canto.org), and calls of *B. hermogenesi* in Núcleo Santa Virgínea, Parque Estadual da Serra do Mar, municipality of São Luiz do Paraitinga, São Paulo, can be heard in a recording of *E. indigoticus* (XC253045; Table 1).

We underscore the importance of continuous scrutiny of the distribution and advertisement call analysis of *B. sulfuratus* and *B. hermogenesi*. The advertisement calls of *B. hermogenesi* need to be redescribed (see Pie et al., 2018b: 12) and a better understanding of the geographical limits between this species and *B. sulfuratus* can elucidate distribution patterns and potentially detect cases of sympatry. To date, the occurrence of *B. hermogenesi* and *Brachycephalus* sp. (other than *B. sulfuratus* and *B. hermogenesi*) at Corcovado, São Paulo, is the only confirmed case of sympatry between species of *Brachycephalus* in the same group. Other cases of sympatry include *Brachycephalus* from distinct groups (*B. pernix* and *B. didactylus* groups and *B. ephippium* and *B. didactylus* groups; Bornschein et al. (2016a) and Bornschein, Pie & Teixeira (2019)). Even in sympatry, the differences between the calls of *B. hermogenesi* and *Brachycephalus* sp. and between *B. hermogenesi* and *B. sulfuratus* are substantial and could provide pre-zygotic isolation. Although some species in the *B. ephippium* group are additively insensitive to the own advertisement call (Goutte et al., 2017), which would suggest loss of active selection pressure and variation maintained by inertia, it must be considered that this scenario may not apply to the other groups (Monteiro et al., 2018b) and, also, that the species may actively perceive call emissions through vibrations in other body receptors.

## Conclusions

*Brachycephalus sulfuratus* differs from *B. hermogenesi* only by its advertisement calls; other morphological characters previously suggested to distinguished *B. sulfuratus* from *B. hermogenesi* are extremely variable and show overlap between these two species. The advertisement calls of these species differ greatly from each other and can be easily recognized by the human ear in the field. *Brachycephalus sulfuratus* presents few notes per call with only isolated notes and *B. hermogenesi* present high number of notes per call with isolated notes and note groups. The advertisement calls of *B. sulfuratus* resemble those of species of the *B. ephippium* species group, whereas the calls of *B. hermogenesi* resemble those of the *B. pernix* species group. Understanding the evolution of these advertisement calls should require a more in-depth investigation.

All previous records of *B. hermogenesi* from southern Brazil should instead be considered as *B. sulfuratus*, in a possibly cascading error resulting from the inadequate revision of the records prior to the description of *B. sulfuratus* (Condez et al., 2016). A large region in the south of the state of São Paulo needs to be further investigated to confirm the presence of *B. hermogenesi*; the previous records were reverted to *Brachycephalus* sp. *Brachycephalus sulfuratus* is distributed much further north than previously thought and it is possible that sympatry with *B. hermogenesi* may occur in the southeast of the city of São Paulo. This region in the southeast of São Paulo is particularly interesting because many species of different taxa have their range limits there. The biogeographic explanation of this pattern seems to be limited to the past distribution of forest patches, which could have been previously isolated and are now distributed continuously, allowing possible secondary contact of species.

The *B. hermogenesi* type series possibly includes a second species of flea toad, not yet identified. This situation involves a locality of a *B. hermogenesi* paratype, and probably not the holotype. Therefore, there is no evidence, at this moment, to suspect the name *B. hermogenesi* as a possible synonym for another *Brachycephalus* species, as *B. didactylus*, for example. It is necessary to deepen the field studies to identify the local populations and to clarify the limits of the geographic distribution, as well as to review the identification of museum material, including the type series of *B. hermogenesi*.

Phylogenetic analysis provided evidence that at least *B. sulfuratus* probably includes more than one species under this name, although this species, as presently defined, has a similar calling pattern in its wide geographical distribution, from southeastern São Paulo to Santa Catarina (Table 1; Fig. 6). In parallel, our *B. hermogenesi* call analyses provided the first association of a call pattern across the geographic distribution of this species (Table 1; Fig. 6), but this does not mean that only one species is necessarily included under this name, because distinct species of *Brachycephalus* may have indistinct calls (Pie et al., 2018b). Combined with the fact that the *B. didactylus* group includes cryptic species, difficult or even impossible to identify in preservative, that occur or can occur locally in sympathy, we recommend a solid and broad review of the taxonomy of the group based on own analyses of large series of specimens and calls.

## Appendix 1

Advertisement calls analyzed in the present study. Abbreviation: MHNCI = Museu de História Natural Capão da Imbuia, Curitiba, Paraná.

***Brachycephalus sulfuratus***. SÃO PAULO: Base of the Serra Água Limpa, municipality of Apiaí MHNCI 129; Biquinha, municipality of Juquiá MHNCI 128; near the Jurupará dam, municipality of Piedade MHNCI 123–5; Núcleo Itutinga-Pilões, Parque Estadual da Serra do Mar, municipality of Cubatão MHNCI 126–7; Serra do Guaraú, on the border of the municipalities of Cajati and Jacupiranga MHNCI 130; Torre Embratel, municipality of Cajati MHNCI 218. PARANÁ: Caratuval, near the Parque Estadual das Lauráceas, municipality of Adrianópolis MHNCI 131; Caratuval, Parque Estadual das Lauráceas, municipality of Adrianópolis MHNCI 132; Entroncamento Teba, Rio Turvo, municipality of Campina Grande do Sul MHNCI 219; Fazenda Thalia, municipality of Balsa Nova MHNCI 134; Morro do Canal, municipality of Piraquara MHNCI 220; Reserva Particular do Patrimônio Natural Salto Morato, municipality of Guaraqueçaba MHNCI 133. SANTA CATARINA: Monte Crista, municipality of Garuva MHNCI 221; Morro do Garrafão, municipality of Corupá MHNCI 137; Morro Garuva, municipality of Garuva MHNCI 136; Serra do Pico, municipality of Joinville MHNCI 217; Truticultura, municipality of Garuva MHNCI 135.

***Brachycephalus hermogenesi***. SÃO PAULO: Corcovado, municipality of Ubatuba MHNCI 166; Estação Biológica de Boracéia, municipality of Salesópolis MHNCI 166–9; Morro do Cantagalo, municipality of Caraguatatuba MHNCI 222–3; Núcleo Cunha, Parque Estadual da Serra do Mar, municipality of Cunha MHNCI 170–1; Núcleo Picinguaba, Parque Estadual da Serra do Mar, municipality of Ubatuba MHNCI 172–87; Parque Natural Municipal Nascentes de Paranapiacaba, municipality of Santo André MHNCI 213–6; Trilha do Ipiranga 50 m from the Rio Ipiranga, Núcleo Santa Virgínia, Parque Estadual da Serra do Mar, municipality of São Luiz do Paraitinga MHNCI 188–92.

***Brachycephalus* sp. (other than *B. sulfuratu*s and *B. hermogenesi*)**. RIO DE JANEIRO: Trilha do Corisco, municipality of Paraty MHNCI 206–12. SÃO PAULO: Corcovado, municipality of Ubatuba MHNCI 193–205.

## Supplemental Information

10.7717/peerj.10983/supp-1 Supplemental Information 1 Accession numbers and corresponding information on all 12S and 16S sequences of specimens of the *Brachycephalus didactylus* species group on GenBank. Abbreviation: NA = not available.

10.7717/peerj.10983/supp-2 Supplemental Information 2 MHNCI 124 Brachycephalus sulfuratus. MHNCI 124 Brachycephalus sulfuratus near Jurupara dam voucher MHNCI 10791 or MHNCI 10792 29 set 2016 MRBornschein.

10.7717/peerj.10983/supp-3 Supplemental Information 3 MHNCI 124 Brachycephalus sulfuratus. MHNCI 124 Brachycephalus sulfuratus near Jurupara dam voucher MHNCI 10791 or MHNCI 10792 29 set 2016 MRBornschein cutted.

10.7717/peerj.10983/supp-4 Supplemental Information 4 MHNCI 125 Brachycephalus sulfuratus. MHNCI 125 Brachycephalus sulfuratus near Jurupara dam not collected 29 set 2016 MRBornschein.

10.7717/peerj.10983/supp-5 Supplemental Information 5 MHNCI 126 Brachycephalus sulfuratus. MHNCI 126 Brachycephalus sulfuratus Nucleo Itutinga Piloes ind 1 not collected 08 out 2016 MRBornschein.

10.7717/peerj.10983/supp-6 Supplemental Information 6 MHNCI 127 Brachycephalus sulfuratus. MHNCI 127 Brachycephalus sulfuratus Nucleo Itutinga Piloes ind 2 not collected 08 out 2016 MRBornschein.

10.7717/peerj.10983/supp-7 Supplemental Information 7 MHNCI 128 Brachycephalus sulfuratus. MHNCI 128 Brachycephalus sulfuratus Biquinha not collected 18 set 2016 MRBornschein.

10.7717/peerj.10983/supp-8 Supplemental Information 8 MHNCI 129 Brachycephalus sulfuratus. MHNCI 129 Brachycephalus sulfuratus Serra Agua Limpa MHNCI 11583 26 out 2011 MRBornschein.

10.7717/peerj.10983/supp-9 Supplemental Information 9 MHNCI 130 Brachycephalus sulfuratus. MHNCI 130 Brachycephalus sulfuratus Serra do Guarau not collected 22 out 2016 MRBornschein.

10.7717/peerj.10983/supp-10 Supplemental Information 10 MHNCI 131 Brachycephalus sulfuratus. MHNCI 131 Brachycephalus sulfuratus Caratuval near Parque Estadual Lauraceas MHNCI 11571 L Correa.

10.7717/peerj.10983/supp-11 Supplemental Information 11 MHNCI 132 Brachycephalus sulfuratus. MHNCI 132 Brachycephalus sulfuratus Caratuval Parque Estadual Lauraceas not collected 18 dez 2010 L Correa.

10.7717/peerj.10983/supp-12 Supplemental Information 12 MHNCI 133 Brachycephalus sulfuratus. MHNCI 133 Brachycephalus sulfuratus Reserva Particular do Patrimonio Natural Salto Morato not collected 10 marco 2016 MRBornschein.

10.7717/peerj.10983/supp-13 Supplemental Information 13 MHNCI 134 Brachycephalus sulfuratus. MHNCI 134 Brachycephalus sulfuratus Fazenda Thalia not collected MRBornschein.

10.7717/peerj.10983/supp-14 Supplemental Information 14 MHNCI 135 Brachycephalus sulfuratus. MHNCI 135 Brachycephalus sulfuratus Truticultura not collected MRBornschein.

10.7717/peerj.10983/supp-15 Supplemental Information 15 MHNCI 136 Brachycephalus sulfuratus. MHNCI 136 Brachycephalus sulfuratus Morro Garuva not collected 02 nov 2016 Andre Confetti.

10.7717/peerj.10983/supp-16 Supplemental Information 16 MHNCI 137 Brachycephalus sulfuratus. MHNCI 137 Brachycephalus sulfuratus Morro do Garrafão not collected LFRibeiro.

10.7717/peerj.10983/supp-17 Supplemental Information 17 MHNCI 165 Brachcephalus hermogenesi Corcovado. MHNCI 165 000107 0122S4 Brachy hermogenesi Corcovado specimen one two starting notes lost without interruption.

10.7717/peerj.10983/supp-18 Supplemental Information 18 MHNCI 168 *Brachycephalus hermogenesi*. MHNCI 168 190330 05 Brachy hermogenesi ind 03 Boraceia 30 marco 2019 MRB.

10.7717/peerj.10983/supp-19 Supplemental Information 19 MHNCI 169 Brachycephlus hermogenesi. MHNCI 169 190330 06 Brachy hermogenesi ind 04 Boraceia 30 marco 2019 MRB.

10.7717/peerj.10983/supp-20 Supplemental Information 20 MHNCI 172 Brachycephalus hemogenesi Núcleo Picinguaba. MHNCI 172 BLR00061 Brachy hemogenesi Picinguaba ind 01 ex 01 sem detalhes 13 dez 2017 MRB.

10.7717/peerj.10983/supp-21 Supplemental Information 21 MHNCI 173 *Brachycephalus hermogenesi* Núcleo Picinguaba. MHNCI 173 BLR00062 B hermogenesi Picinguaba ind2 ex1 4 notas perdidas 13dez2017 MRB.

10.7717/peerj.10983/supp-22 Supplemental Information 22 MHNCI 174 *Brachycephalus hermogenesi* Núcleo Picinguaba. MHNCI 174 BLR00064 B hermogenesi Picinguaba ind3 6 notas perdidas 13dez2017 MRB.

10.7717/peerj.10983/supp-23 Supplemental Information 23 MHNCI 175 Brachecephalus hermogenesi Núcleo Picinguaba. MHNCI 175 BLR00065 B hermogenesi Picinguaba ind1ex2 tudo 13 dez 2017 MRB.

10.7717/peerj.10983/supp-24 Supplemental Information 24 MHNCI 176 *Brachycephalus hermogenesi* Núcleo Picinguaba. MHNCI 176 BLR00067 B hermogenesi Picinguaba ind1ex3 tudo ind4ex1 3 perdidas 13dez2017 MRB.

10.7717/peerj.10983/supp-25 Supplemental Information 25 MHNCI 177 *Brachycephalus hermogenesi* Núcleo Picinguaba. MHNCI 177 BLR00068 B hermogenesi_Picinguaba ind2 ex2 tudo 05cm 13 dez 2017 MRB.

10.7717/peerj.10983/supp-26 Supplemental Information 26 MHNCI 178 *Brachycephalus hermogenesi* Núcleo Picinguaba. MHNCI 178 BLR00069 B hermogenesi Picinguaba ind3 ex2 5 notas perdidas 13 dez 2017 MRB.

10.7717/peerj.10983/supp-27 Supplemental Information 27 MHNCI 179 *Brachycephalus hermogenesi* Núcleo Picinguaba. MHNCI 179 BLR00070 B hermogenesi Picinguaba ind 04 ex 02 13 dez 2017 MRB.

10.7717/peerj.10983/supp-28 Supplemental Information 28 MHNCI 180 *Brachycephalus hermogenesi* Núcleo Picinguaba. MHNCI 180 BLR00071 B hermogenesi Picinguaba ind 03 ex 03 sem detalhes 13 dez 2017 MRB.

10.7717/peerj.10983/supp-29 Supplemental Information 29 MHNCI 181 *Brachycephalus hermogenesi* Núcleo Picinguaba. MHNCI 181 BLR00072 B hermogenesi Picinguaba ind3 ex4 perdeu uma nota 13 dez 2017 MRB.

10.7717/peerj.10983/supp-30 Supplemental Information 30 MHNCI 182 *Brachycephalus hermogenesi* Núcleo Picinguaba. MHNCI 182 BLR00073 B hermogenesi Picinguaba ind2 ex3 soh o final do canto 13 dez 2017 MRB.

10.7717/peerj.10983/supp-31 Supplemental Information 31 MHNCI 183 *Brachycephalus hermogenesi* Núcleo Picinguaba. MHNCI 183 BLR00076 B hermogenesi Picinguaba ind4ex3 tudo 5-20cm distancia 13dez2017 MRB.

10.7717/peerj.10983/supp-32 Supplemental Information 32 MHNCI 184 *Brachycephalus hermogenesi* Núcleo Picinguaba. MHNCI 184 000106 0115S4 Brachy hermogenesi Picinguaba specimen one several starting notes lost without interruption.

10.7717/peerj.10983/supp-33 Supplemental Information 33 MHNCI 185 *Brachycephalus hermogenesi* Núcleo Picinguaba. MHNCI 185 000106 0117S4 Brachy hermogenesi Picinguaba specimen two two starting notes lost without interruption.

10.7717/peerj.10983/supp-34 Supplemental Information 34 MHNCI 186 *Brachycephalus hermogenesi* Núcleo Picinguaba. MHNCI 186 000106 0119S4 Brachy hermogenesi Picinguaba specimen three one starting notes lost without interruption.

10.7717/peerj.10983/supp-35 Supplemental Information 35 MHNCI 187 *Brachycephalus hermogenesi* Núcleo Picinguaba. MHNCI 187 000106 0120S4 Brachy hermogenesi Picinguaba specimen four several starting notes lost without interruption.

10.7717/peerj.10983/supp-36 Supplemental Information 36 MHNCI 213 Brachecephalus hermogenesi Parque Natural Municipal Nascentes de Paranapiacaba. MHNCI 213 080409 02 B hermogenesi ind1 muito perdido ruim Paranapiacaba MRB.

10.7717/peerj.10983/supp-37 Supplemental Information 37 MHNCI 188 *Brachycephalus hermogenesi* Trilha do Ipiranga. MHNCI_188_000103_0135S4_Brachy hermogenesi Trilha do Ipiranga ind 1 e unknown starting notes with interruption.

10.7717/peerj.10983/supp-38 Supplemental Information 38 MHNCI 189 *Brachycephalus hermogenesi* Trilha do Ipiranga. MHNCI 189 000103 0137S4 Brachy hermogenesi Trilha do Ipiranga ind 2 three starting notes lost.

10.7717/peerj.10983/supp-39 Supplemental Information 39 MHNCI 190 *Brachycephalus hermogenesi* Trilha do Ipiranga. MHNCI 190 000103 0140S4 Brachy hermogenesi Trilha do Ipiranga ind 3 three starting notes lost.

10.7717/peerj.10983/supp-40 Supplemental Information 40 MHNCI 191 *Brachycephalus hermogenesi* Trilha do Ipiranga. MHNCI 191 000103 0141S4 Brachy hermogenesi Trilha do Ipiranga ind 4 four starting notes lost.

10.7717/peerj.10983/supp-41 Supplemental Information 41 MHNCI 193 Brachycephalus sp parte final do canto Corcovado. MHNCI 193 BLR00084 Brachy sp ind1 parte final Corcovado 18dez2017 MRB.

10.7717/peerj.10983/supp-42 Supplemental Information 42 MHNCI 194 Brachycephalus sp Corcovado. MHNCI 194 BLR00085 Brachy sp ind2 2m Corcovado 18dez2017 MRB.

10.7717/peerj.10983/supp-43 Supplemental Information 43 MHNCI 195 Brachycephalus sp Corcovado. MHNC 195 BLR00086 Brachy sp ind3 perdeu notas 20cm Corcovado 18dez2017 MRB.

10.7717/peerj.10983/supp-44 Supplemental Information 44 MHNCI 196 Brachycephalus sp Corcovado. MHNCI 196 BLR00087 Brachy sp ind4ex1 perdeu 3 notas 30-100cm Corcovado 18dez2017 MRB.

10.7717/peerj.10983/supp-45 Supplemental Information 45 MHNCI 197 Brachycephalus sp Corcovado. MHNCI 197 BLR00089 Brachy sp ind5 soh final Corcovado 18dez2017 MRB.

10.7717/peerj.10983/supp-46 Supplemental Information 46 MHNCI 198 Brachycephalus sp Corcovado. MHNCI 198 BLR00091 Brachy sp ind6ex1 4 notas perdidas 10-50cm Corcovado 18dez2017 MRB.

10.7717/peerj.10983/supp-47 Supplemental Information 47 MHNCI 199 Brachycephalus sp Corcovado. MHNCI 199 BLR00092 Brachy sp ind6ex2 soh final 50-80cm Corcovado 18dez2017 MRB.

10.7717/peerj.10983/supp-48 Supplemental Information 48 MHNCI 200 Brachycephalus sp Corcovado. MHNCI 200 BLR00093 Brachy sp ind4ex2 perdeu 7 notas 20cm Corcovado 18dez2017 MRB.

10.7717/peerj.10983/supp-49 Supplemental Information 49 MHNCI 201 Brachycephalus sp Corcovado. MHNCI 201 BLR00094 Brachy sp ind7 perdeu 4 notas 20-100cm Corcovado 18dez2017 MRB.

10.7717/peerj.10983/supp-50 Supplemental Information 50 MHNCI 202 Brachycephalus sp Corcovado. MHNCI 202 BLR00095 Brachy sp ind8 perdeu 6 notas Corcovado 18dez2017 MRB.

10.7717/peerj.10983/supp-51 Supplemental Information 51 MHNCI 203 Brachycephalus sp Corcovado. MHNCI 203 000102 0129S4 Brachycephalus sp ind1 Corcovado two starting notes lost.

10.7717/peerj.10983/supp-52 Supplemental Information 52 MHNCI 204 Brachycephalus sp Corcovado. MHNCI 204 000102 0130S4 Brachycephalus sp ind2 Corcovado two starting notes lost.

10.7717/peerj.10983/supp-53 Supplemental Information 53 MHNCI 205 Brachycephalus sp Corcovado. MHNCI 205 000102 0132S4 Brachycephalus sp ind4 ind5 Corcovado two starting notes lost.

10.7717/peerj.10983/supp-54 Supplemental Information 54 MHNCI 211 Brachycephalus sp Trilha do Corisco. MHNCI 211 000104 0113S4 Brachycephalus sp ind1 Trilha do Corisco several starting notes lost.

10.7717/peerj.10983/supp-55 Supplemental Information 55 MHNCI 212 Brachycephalus sp Trilha do Corisco. MHNCI 212 000104 0114S4 Brachycephalus sp ind2 Trilha do Corisco several starting notes lost.

10.7717/peerj.10983/supp-56 Supplemental Information 56 MHNCI 206 Brachycephalus sp Trilha do Corisco. MHNCI 206 BLR00053 Brachycephalus sp Trilha do Corisco ind1 perdeu inicio 40 cm 12dez2017 MRB.

10.7717/peerj.10983/supp-57 Supplemental Information 57 MHNCI 207 Brachycephalus sp Trilha do Corisco. MHNCI 207 BLR00054 Brachycephalus sp Trilha do Corisco ind2 perdeu inicio 12dez2017 MRB.

10.7717/peerj.10983/supp-58 Supplemental Information 58 MHNCI 208 Brachycephalus sp Trilha do Corisco. MHNCI 208 BLR00055 Brachycephalus sp Trilha do Corisco ind3 microfone flutuou 12dez2017 MRB.

10.7717/peerj.10983/supp-59 Supplemental Information 59 MHNCI 209 Brachycephalus sp Trilha do Corisco. MHNCI 209 BLR00056 Brachycephalus sp Trilha do Corisco ind4 20-60 cm 12dez2017 MRB.

10.7717/peerj.10983/supp-60 Supplemental Information 60 MHNCI 217 Brachycephalus sulfuratus Serra do Pico. MHNCI 217 190206 05 Brachycephalus sp mais B sulfuratus Serra do Pico MRB 6fev2019.

10.7717/peerj.10983/supp-61 Supplemental Information 61 MHNCI 218 Brachycephalus sulfuratus Torre Embratel. MHNC 218 BLR00165 Brachy sp mais Brachy sulfuratus Torre Embratel MRB não coletado.

10.7717/peerj.10983/supp-62 Supplemental Information 62 MHNCI 219 Brachycephalus sulfuratus Entroncamento Teba. MHNCI 219 200123 0448S4 Brachycephalus sulfuratus Entroncamento Teba 20 cm from microphone LFR JAN-2020.

10.7717/peerj.10983/supp-63 Supplemental Information 63 MHNCI 220 Brachycephalus sulfuratus Morro do Canal. MHNCI 220 Brachycephalus sulfuratus Morro do Canal 150 cm from microphone LFRibeiro JAN-2017.

10.7717/peerj.10983/supp-64 Supplemental Information 64 MHNCI 221 Brachycephalus sulfuratus Monte Crista. MHNCI 221 161115 02 Brachycephalus sulfuratus Monte Crista 100 cm from microphone Luiz Fernando Ribeiro NOV-2016.

10.7717/peerj.10983/supp-65 Supplemental Information 65 MHNCI 222 *Brachycephalus hermogenesi* ind1 Morro do Cantagalo. MHNCI 222 180802 08 *Brachycephalus hermogenesi* ind1 parte do canto Morro do Cantagalo-Caraguatatuba LC.

10.7717/peerj.10983/supp-66 Supplemental Information 66 MHNCI 223 *Brachycephalus hermogenesi* ind 2 Morro do Cantagalo. MHNCI 223 180802 09 *Brachycephalus hermogenesi* ind2 parte do canto Morro do Cantagalo-Caraguatatuba LC.

## Additional Information and Declarations

### Data Availability

The following information was supplied regarding data availability: Raw recording data is available in Table 2. All specimens are deposited in the collection of the Museu de História Natural Capão da Imbuia, Curitiba, Paraná, Brazil (MHNCI 123-137, MHNCI 165-223). Recordings are available in the Supplemental Files.
