# Supplementary material for: A review of the diagnosis and geographical distribution of the recently described flea toad Brachycephalus sulfuratus in relation to B. hermogenesi (Anura: Brachycephalidae)
Source: PeerJ. 2021 Mar 4;9:e10983. doi: 10.7717/peerj.10983 (PMC7937348; doi:10.7717/peerj.10983)
Supplement: Supplemental Information 1 — Abbreviation: NA = not available. [file peerj-09-10983-s001.docx]

## Table S1. Accession numbers and corresponding information on all 12S and 16S sequences of specimens of the *Brachycephalus didactylus* species group on GenBank. Abbreviation: NA = not available.

| 12S accession number | 16S accession number | Locality, according to GenBank | Voucher information on GenBank | Tip label on phylogeny (Fig. 7) |
| --- | --- | --- | --- | --- |
| HQ435682 | NA | Piedade, SP | CFBH 12908 | *“B. hermogenesi*”; Municipality of Piedade, São Paulo |
| NA | MT929508 | NA | TC134 | “*B. hermogenesi*”; without locality |
| NA | MT929507 | NA | TC133 | “*B. hermogenesi*”; without locality |
| MT901272 | MT929506 (MK697372, MG889426) | NA | TC129 | “*B. hermogenesi*”; without locality |
| MT901271 | MT929505 | NA | TC127 | “*B. hermogenesi*”; without locality |
| NA | MK697374 | Picinguaba, SP | CFBH 29793 | “*B. hermogenesi*”; Núcleo Picinguaba, São Paulo |
| NA | MK697373 | Paraibuna, SP | AAG-UFU 177 | “*B. hermogenesi*”; Municipality of Paraibuna, São Paulo |
| HQ435682 | NA | Piedade, SP | CFBH 12908 | “*B. hermogenesi*”; Municipality of Piedade, São Paulo |
| NA | KU321531 | Picinguaba, SP | CFBH29794 | “*B. hermogenesi*”; Núcleo Picinguaba, São Paulo |
| KX198033 | KX025330 | Quatro Barras, PR | Corvo | “*B. sulfuratus*”; Corvo, Paraná |
| KX198032 | NA | Balsa Nova, PR | Fazenda Thalia | “*B. sulfuratus*”; Fazenda Thalia, Paraná |
| KX198031 | KX025307 | Caratuval | Caratuval | “*B. sulfuratus*”; Caratuval, near the Parque Estadual das Lauráceas, Paraná |
| KX198030 | NA | Apiaí, SP | Apiai | “*B. sulfuratus*”; Base of the Serra Água Limpa, São Paulo |
| MK697441 | KU321532 (MK697392) | São Francisco do Sul, SC | CFBH39141 | “*B. sulfuratus*”; Morro do Cantagalo, Santa Catarina |
| NA | KU321535.1 | Cananéia, SP | CFBH39149 | “*B. sulfuratus*”; Morro do Cantagalo, Santa Catarina |
| NA | KU321534 | Quatro Barras, PR | CFBH39150 | “*B. sulfuratus*”; Morro Anhangava, Paraná |
| MK697439 | KU321533 | Joinville, SC | CFBH39146 | “*B. sulfuratus*”; Castelo dos Bugres, Santa Catarina |
| MK697440 | MK697391 | Quatro Barras, PR | CFBH40436 | “*B. sulfuratus*”; Morro Anhangava, Paraná |
| MK697438 | MK697389 | Barra do Turvo, SP | CTMZ 2970 | “*B. sulfuratus*”; Municipality of Barra do Turvo, São Paulo |
| MK697437 | MK697388 | Cananéia, SP | CFBH 39148 | “*B. sulfuratus*”; Parque Estadual da Ilha do Cardoso, São Paulo |
| NA | MG889430 | Itapoa, SC | JPCM-389 | “*B. sulfuratus*”; Braço do Norte, Santa Catarina |
| NA | MG889429 | Joinville, SC | TC-244 | “*B. sulfuratus*”; “Joinville”, Santa Catarina |
| NA | MG889428 | Quatro Barras, PR | TC-141 | “*B. sulfuratus*”; “Quatro Barras”, Paraná |
| MT901270 | MT929504 | NA | MNRJ37327 | “*B. didactylus*”; without locality |
| MT901268 | MT929503 | NA | MNRJ68818 | “*B. didactylus*”; without locality |
| MT901267 | NA | NA | CFBH11506 | “*B. didactylus*”; without locality |
| MT901269 | NA | NA | MNRJ37326 | “*B. didactylus*”; without locality |
| MK697417 | NA | Cachoeiras do Macacu, RJ | MNRJ 54402 | “*B. didactylus*”; “Cachoeiras do Macacu”, Rio de Janeiro |
| HQ435678 | HQ435692 | Ilha Grande, RJ | CFBH 12907 | “*B. didactylus*”; “Ilha Grande”, Rio de Janeiro |
